# A Validation Study of a High-Throughput Sequencing Workflow for the Detection of Grapevine Viruses and Viroids

**DOI:** 10.3390/pathogens15090998

**Published:** 2026-09-21

**Authors:** Kankana Ghoshal, James Phelan, Yahya Z. A. Gaafar, Ian Boyes, Reece Hoffmann, Michael Rott

**Affiliations:** Centre for Plant Health, Canadian Food Inspection Agency, 8801 East Saanich Rd, North Saanich, BC V8L 1H3, Canada; konkana2003@gmail.com (K.G.); yahya.gaafar@inspection.gc.ca (Y.Z.A.G.); ian.boyes@inspection.gc.ca (I.B.);

**Keywords:** plant viruses, diagnostics, method, validation, HTS

## Abstract

Grapevine (*Vitis vinifera* L.) hosts a diverse range of viruses and viroids that differ in genome type, abundance, and distribution, creating challenges for reliable detection. High-throughput sequencing (HTS) is a powerful non-targeted tool for pathogen diagnostics, but its routine implementation requires validated workflows and objective interpretation criteria. Here, we present a validation framework for HTS-based plant virus and viroid diagnostics and demonstrate its application using an in-house grapevine pathogen detection workflow. Ten composite reference samples containing 85 virus and viroid occurrences were analyzed across a dilution series to evaluate diagnostic accuracy, detection limits, repeatability, reproducibility, and contamination risk. Results showed that sequencing quality metrics alone were insufficient for diagnostic interpretation. Additional indicators, including mapped reads and internal control depth, were necessary to assess sample suitability and define minimum performance requirements. Viral detection was evaluated using genome coverage and relative abundance (weight) metrics. A positive relationship between these parameters enabled the establishment of quantitative detection thresholds and a three-tier classification system: positive, likely positive, and likely negative. Diagnostic accuracy decreased with dilution because of reduced sensitivity for low-abundance pathogens, whereas repeatability and within-laboratory reproducibility remained high and contamination signals stayed below detection thresholds. Although the performance metrics are workflow-specific, this study provides a practical framework for laboratory validation of HTS-based plant virus and viroid diagnostics.

## 1. Introduction

Grapevine (*Vitis vinifera* L.) is susceptible to infection by a wide diversity of virus and viroids representing multiple genera and genome types worldwide [1]. These pathogens can significantly impact vine health, fruit quality, and yield, making their accurate detection essential for certification, surveillance, and management programs. Traditional diagnostic methods, such as enzyme-linked immunosorbent assay (ELISA) and polymerase chain reaction (PCR/qPCR), rely on targeted detection of known pathogens. In contrast, biological indexing or bioassays are less targeted and can detect a broader range of viruses, including novel or previously uncharacterized isolates, based on symptom expression in indicator plants. However, bioassays are time-consuming, labour-intensive, and can be variable in sensitivity and reproducibility depending on host–pathogen interactions and environmental conditions [2]. Overall, conventional methods remain limited either by their dependence on prior knowledge of the target organism or by practical constraints that reduce scalability and consistency.

In contrast, high-throughput sequencing (HTS) provides a non-targeted, comprehensive approach capable of capturing the full spectrum of nucleic acids present in a sample [3]. This enables simultaneous detection of known viruses, genetic variants, and previously uncharacterized pathogens [4]. As a result, HTS offers equal or greater inclusivity than that of conventional molecular, serological, and bioassay-based methods. Combined with increasing cost efficiency, scalability, and reduced turnaround time, HTS is transitioning from a research-focused tool to a practical platform for routine plant virus diagnostics in regulatory and certification laboratories [5,6,7].

Despite these advantages, the implementation of HTS in routine Phytovirus diagnostics requires the establishment of rigorous, transparent, and reproducible validation frameworks. This need arises because HTS-based assays differ fundamentally from traditional diagnostic methods in both data generation and result interpretation. In conventional assays, a positive result is defined using clear, quantitative, and widely accepted thresholds, such as cycle threshold (Ct) values in real-time PCR, amplicon size in gel-based PCR assays, or optical density measurements in ELISA [8]. These criteria are relatively straightforward to interpret and supported by decades of methodological refinement. In contrast, HTS produces large, complex datasets that require multi-step analytical workflows, making the determination of what constitutes a “positive” detection less direct and more context-dependent. The interpretation of HTS data is influenced by numerous technical and analytical variables, each of which can significantly affect the outcome. These include factors such as sample quality and nucleic acid extraction efficiency, library preparation methods, sequencing platform characteristics, and sequencing depth. In addition, the choice of enrichment strategy plays a critical role in shaping the dataset. Approaches such as total nucleic acid (TNA), total RNA sequencing, ribodepleted RNA, small RNA sequencing, or double-stranded RNA (dsRNA) enrichment are designed to target different components of the virome and can therefore yield datasets that can differ in composition, sensitivity, and bias. A laboratory analyzing the same sample using different enrichment strategies may obtain divergent detection results [9].

Further complexity arises at the bioinformatics stage, where raw sequence data are processed and interpreted. A wide range of analytical pipelines are currently available, including tools such as Virtool, VirusDetect, Viroscope, and PVDP (https://www.virtool.ca [10], https://viroscope.io/ [11]), last accessed on 25 December 2025. These platforms differ in their underlying algorithms, reference databases, filtering criteria, and output formats. Consequently, they may produce varying lists of detected viruses, along with different metrics for confidence or abundance. Interpreting these outputs requires expertise and, in many cases, subjective judgement, particularly when dealing with low read counts, partial genome assemblies, or sequences with limited similarity to known viruses. The lack of harmonized reporting standards further complicates cross-study comparisons.

Taken together, these factors highlight that, unlike traditional assays, HTS-based diagnostics do not yet rely on universally accepted criteria for defining positive or negative results. Instead, detection thresholds and decision rules are often determined on a case-by-case basis, leading to variability in interpretation across laboratories and studies. This subjectivity, combined with inconsistent application of analytical criteria, underscores the urgent need for standardized guidelines, validation metrics, and performance benchmarks to ensure reliability, reproducibility, and regulatory acceptance of HTS in routine Phytovirus diagnostics [12].

A key challenge in HTS diagnostics is distinguishing true low-titre infections from background noise, cross-contamination, and bioinformatic artefacts. Because HTS is inherently a “catch-all” method, weak signals from non-target organisms or environmental contaminants can complicate interpretation. This highlights the critical need for well-defined validation metrics and decision thresholds that can reliably differentiate true infections from false positives and false negatives. Establishing such criteria is essential to ensure diagnostic accuracy, reproducibility, and confidence in HTS-based results, particularly in regulated plant health applications.

To address these challenges, various laboratories have developed diverse HTS workflows and analytical strategies tailored to specific plant systems and diagnostic needs. A validation study using an HTS assay for grapevine virus diagnostics evaluates two template extraction methods (TNA and dsRNA) across seasonal tissue types (spring petioles vs. fall dormant canes) [13]. The results showed that TNA extraction is a more practical and efficient alternative to dsRNA, particularly for viroid detection in fall canes. In contrast, the dsRNA extraction method exhibited slightly higher sensitivity and specificity. Using a positive detection threshold of >10 reads, the assay revealed that combining results from different tissue types and sampling seasons increased diagnostic sensitivity to 100% [13].

A ribodepleted total RNA and Illumina sequencing HTS protocol along with validation performance criteria for routine virus indexing of Musa (banana) germplasm, incorporating a unique “alien control” (BYDV-infected wheat) to monitor cross-contamination, was developed and used to establish adaptive detection thresholds. The study found that, although HTS provides superior inclusivity and sensitivity than standard PCR-based methods, expert interpretation and secondary Immunocapture-PCR testing are required to address the unique biological challenge of differentiating active viral infections from integrated endogenous viral elements [14].

An interlaboratory validation was conducted across three U.S.-based laboratories to evaluate the reproducibility and diagnostic sensitivity of a standardized ribodepleted total RNA HTS protocol for detecting a panel of 33 viruses and six viroids in apple leaf lamina, grapevine petioles and dormant canes, and *Prunus* petioles and budwood. The authors recommended a minimum of 15 million reads per sample to achieve a 100% target detection rate. Using diagnostic thresholds of at least 20 reads and 20% genome coverage, the assay achieved diagnostic sensitivities ranging from 94% to 100% across host species and outperformed conventional and real-time PCR assays [15].

Porcher et al. [16] validated an in-house ribodepleted total RNA HTS workflow for official plant health controls, demonstrating the simultaneous detection of RNA viruses, DNA viruses, and viroids in challenging plant matrices such as *Vitis* and *Euphorbia*. A deep learning tool (VirHunter) was incorporated into the bioinformatics pipeline to facilitate the discovery of novel or uncharacterized pathogens. Analytical sensitivity and repeatability were evaluated using three specific target pathogens: tomato brown rugose fruit virus, tomato leaf curl New Delhi virus, and pepper chat fruit viroid. Based on this analysis, the authors established a detection threshold of at least 10 reads, a 250 bp contig, and 33% consecutive genome coverage for positive detection. However, there remains a lack of harmonized, quantitative frameworks that integrate sequencing quality metrics with biologically meaningful thresholds for pathogen detection. In particular, standardized approaches that link viral abundance and genome coverage to detection confidence are still limited.

In this study, we developed and validated a quantitative framework for HTS-based detection of grapevine viruses and viroids using a panel of ten composite reference samples containing known virus populations. By systematically evaluating the relationship between viral genome coverage and relative abundance (weight), we established objective thresholds and a three-tier diagnostic decision framework capable of distinguishing true infections from background noise. In addition, we defined key performance metrics and quality control parameters across the entire HTS workflow, including sequencing adequacy, contamination assessment, and reproducibility. Overall, this work provides a step-by-step validation strategy and practical guidance for implementing HTS in plant virus diagnostics. It highlights both the strengths and limitations of the technology and offers a robust framework to support its reliable use in routine testing, while emphasizing the importance of evidence-based threshold determination and risk management in HTS-based pathogen detection systems.

## 2. Materials and Methods

For a detailed protocol of the HTS method as it is designed to be performed, see Supplementary Documents S1, HTS Test Method for Plant Viruses and Viroids, Supplementary Document S2 dsRNA Enrichment for HTS Using the Hamilton Nimbus Presto and Supplementary Document S3 HTS Library Preparation from dsRNA Using the Hamilton MICROLAB^®^ STAR™ (Hamilton Company, Reno, NV, USA).

### 2.1. Sample Collection and Preparation

To ensure sample standardization and consistency, leaf tissue was harvested in bulk from individual plants, freeze-dried, ground to a fine powder, and thoroughly mixed prior to generating the various dilutions. In addition, to improve sample uniformity, large veins and petioles were removed after grinding, as these tissues were difficult to homogenize and distribute evenly across samples, which could affect the detection of low-titre, phloem-limited viruses.

Eighteen different grapevines and one *Ribes* plant, from the virus-positive collection maintained at the Canadian Food Inspection Agency’s Centre for Plant Health, in British Columbia, Canada, and one healthy control, which have been tested by PCR, ELISA, bioassays and HTS for grapevine viruses, were used to create the samples. The 10 samples, named as virus composite (VC), were created as composites of leaf material from 1–3 virus-infected plants out of 18 positive plants (Table 1), with varying amounts of healthy leaf material to make the different dilutions. All the viruses belong to 9 families and 16 genera (Table 2). Preparation of the reference internal control *Phaseolus vulgaris* cv. Black Bean Turtle Soup (BBTS) infected with Phaseolus vulgaris endornavirus 1 (PvEV-1) is described in Supplementary Document S2 dsRNA Enrichment for HTS Using the Hamilton Nimbus Presto.

### 2.2. DsRNA Enrichment, HTS Libraries and Illumina Sequencing

DsRNA was extracted from plant tissue using an automated protocol based on Rott et al. [17]. Briefly, plant tissue samples, in batches of 48, were transferred to 5 mL collection tubes and homogenized with an 8 mm steel ball using a GenoGrinder2010 (SPEX SamplePrep, Metuchen, NJ, USA) together with a spiked control, BBTS. Then they were centrifuged to pellet debris and placed into the Hamilton Nimbus Presto (Hamilton Company, Reno, NV, USA) for dsRNA extraction (see Supplementary Document S2: dsRNA Enrichment for HTS Using the Hamilton Nimbus Presto). Following dsRNA enrichment, extracts were evaluated using a PCR test to PvEV-1 [18], before being transferred to the Hamilton NGS STAR (Hamilton Company, Reno, NV, USA) to synthesis the HTS libraries (see Supplementary Document S3: HTS Library Preparation from dsRNA Using the Hamilton MICROLAB^®^ STAR™).

Libraries were quantified using Qubit flex fluorometer (Thermofisher Scientific, Waltham, MA, USA) according to the manufacturer’s instructions, normalized and sequenced on a NextSeq1000, using the P2 XLEAP-SBS™ Reagent Kit (100 Cycles) (Illumina Canada Ulc., Vancouver, BC, Canada), to generate 100 base single ended reads.

### 2.3. Data Handling and Analysis

The Illumina sequencing quality assurance parameters used were those provided by Illumina for the NextSeq1000 (Illumina, Inc., San Diego, CA, USA). With ≥90% of bases at Q30 or higher, there was ≥60% cluster occupancy and quality through % Clusters Passing Filter (PF), plus a total reads output for a P2 1 × 100 flow cell of approximately 400 million reads with a total data output of approx. 40 Gb.

Demultiplexed reads were imported into Virtool (https://www.virtool.ca, last accessed on 25 December 2025) and evaluated using three relevant tools available in FastQC 0.11.9 (https://www.bioinformatics.babraham.ac.uk/projects/fastqc, last accessed on 25 December 2025). (1) Quality Distribution at Read Position with median values of <25 or a lower quartile of <10 was considered cause for concern. (2) Nucleotide Composition at Read Positions, where abnormally high G content can indicate a high proportion of adapter dimers. (3) Read-wise quality occurrence is used to check if there is a subset of reads with universally poor quality. Sample sequence files were analyzed using the modified Pathoscope [19] workflow in Virtool (https://www.virtool.ca, last accessed on 25 December 2025). The ref-builder/plant-viruses 0.1.1 plant viral genome reference set (https://github.com/ref-builder/plant-viruses, last accessed on 25 December 2025) was used as a mapping reference for Pathoscope.

For each sample the total number of reads and total reads mapped to the virus database were recorded. For each virus in a sample the following was recorded: weight, the fraction of reads mapped to the virus out of the total number of reads mapped to all viruses in the database; coverage, the fraction of the virus genome that was sequenced; and depth, the mean number of times the whole virus genome was sequenced.

### 2.4. Calculation for Validation Metrics

To comprehensively evaluate diagnostic performance in the validation experiment, six key metrics were calculated: (1) accuracy, (2) specificity, (3) recall, (4) false-positive rate (FPR), (5) precision, and (6) F1 score. These metrics summarize the classification outcomes of viral detection, including true positives (TP), true negatives (TN), false positives (FP), and false negatives (FN). Accuracy was defined as the proportion of correctly classified samples among all samples (i.e., both positive and negative). Specificity measured the ability of the assay to correctly identify true negatives. Recall, also referred to as sensitivity or the true-positive rate, quantified the proportion of actual positives correctly identified as positive. FPR represented the proportion of actual negatives that were incorrectly classified as positive. Precision was defined as the proportion of predicted positive results that were true positives. Finally, the F1 score, calculated as the harmonic mean of precision and recall, provided a balanced measure of positive class performance.

The formulas to determine the validation metrics are the following:

$$specificity=\frac{TN}{TN+FP}$$

$$accuracy=\frac{\left(TP+TN\right)}{\left(TP+TN+FP+FN\right)}$$

$$recall=\frac{\left(TP\right)}{\left(TP+FN\right)}\times 100\%$$

$$FPR=\frac{\left(FP\right)}{\left(FP+TN\right)}$$

$$precision=\frac{\left(TP\right)}{\left(TP+FP\right)}$$

$$F1\;score=\;2\times \frac{\left(precision\times recall\right)}{\left(precision+recall\right)}$$

### 2.5. Repeatability

One-Way ICC and Two-Way (ICC3.1) were calculated using the statsmodels Python package.

### 2.6. Intermediate Precision or Within-Laboratory Reproducibility

Agreement between Analyst 1 and Analyst 2 was assessed using Bland–Altman analysis (standard difference mode) with 95% limits of agreement [20]; 49 paired measurements were analyzed. Mean bias and limits of agreement are reported with 95% confidence intervals. Proportional bias was assessed by linear regression of differences on means. Normality of differences was tested using the Shapiro–Wilk test. Analysis was performed using the ConductScience Bland–Altman Analyzer (v1.0.0).

### 2.7. Monitoring Error/Background Contamination with the Internal Control

For contamination monitoring, percent reads mapped and virus weight were calculated for each detected virus. These calculations focused on both the well with the highest contamination and the median value across all contaminated wells. The applied formula is expressed mathematically here:

$$\%\;Relative\;Contamination=100\%\times \frac{\left(\%\mathrm{RMC}\times Wc\right)}{\left(\%\mathrm{RMi}\times Wi\right)}$$

where %RMc = percent reads mapping to all viruses in the contaminated well; %RMi = percent reads mapping to all viruses in the infected well; Wc = weight of contaminating virus in contaminated well; and Wi = weight of the contaminating virus in an infected well.

### 2.8. Data Processing and Visualization Pipeline

Initial data processing was first done using an excel file. Complex data analysis and data visualization were conducted using the Anaconda Distribution (Python v3.8.5) within a Jupyter Notebook (v6.1.4) interactive environment. Customized scripts were used to run data processing and visualization packages within the Jupyter environment and raw data in the form of CSV files was used as an input for processing. The main python packages used for data processing and core statistical visualizations were generated using Pandas (v1.1.3), SciPy (v1.5.2), NumPy (v1.19.2) Matplotlib (v3.3.2), statsmodels, and Seaborn (v0.11.0). During the development of the scripts, occasionally Google Gemini v3.1 was used. The data that support the findings of this study are available from the corresponding author upon reasonable request.

## 3. Results

### 3.1. Experimental Outline

To capture viral genomic diversity, infected plant material was selected to include 30 viruses with positive-sense single-stranded RNA genomes, one virus with a negative-sense single-stranded RNA genome, one virus with a double-stranded RNA genome, one with a single-stranded DNA genome, one with a double-stranded DNA genome, and four single-stranded circular RNA viroids (Table 2). To limit the number of test samples to a manageable size, two or even three infected plants were combined to create the initial (undiluted) virus composite (VC) sample. Dilutions were prepared using healthy grapevine tissue to mimic a composite test sample and to facilitate the determination of detection thresholds.

Ten VC sample sets (VC01–VC10), each comprising six samples representing healthy (0:1, or infected:total), infected (1:1, or undiluted), and serial dilutions of 1:10, 1:25, 1:50 and 1:100 (10-, 25-, 50- and 100-fold dilutions), were used to evaluate and validate the HTS method. It should be noted that the effective dilution of individual viruses and viroids may be two-/threefold greater, depending on the number of infected plants initially combined to create the undiluted VC sample (Table 1). For VC02–V06, VC09 and VC10, each sample was initially tested in duplicate by two independent analysts (Table 3), with Analyst 1 performing replicates a and b and Analyst 2 performing replicates c and d (experiments 1–4).

Sample cross-contamination was evaluated in experiment 5, which included three undiluted samples, two infected with tomato black ring virus (TBRV) and VC07, together with 45 uninfected samples (Table 3).

Analysis of the data from test runs 1–4, revealed an elevated HTS library failure rate associated with the G lane of the Hamilton NGS STAR. Investigation identified inconsistent sealing of the corresponding CORE I pin, resulting in variability in liquid handling performance. The instrument was subsequently serviced, and the pins upgraded to CORE II. Libraries that failed quality control were excluded from further analysis; however, their inclusion increased the overall percentage library failure rate. Two additional experiments (6 and 7) were conducted using the sample sets VC01–VC10 to generate further validation data. Samples from VC01 and VC07 were tested in triplicate (replicates a, b, and g or c, d, and h) for the undiluted and 10-fold dilutions and in duplicate for the remaining dilutions by two independent analysts. Samples from VC02–VC06 and VC08–VC10 were tested in a single replicate (g or h) for the undiluted and 10-fold dilutions by the same analysts (Table 3). Data from experiments 1–4, 6 and 7 were used to establish thresholds for sample data suitability and performance metrics. Data from experiment 5 were used specifically to assess sample cross-contamination.

### 3.2. DsRNA Enrichment and qPCR Analysis

Double-stranded (dsRNA) enrichment was performed in batches of 48 samples. To assess the consistency and success of the enrichment process across all 336 extractions, a real-time quantitative PCR assay targeting the spiked internal control, BBTS, was employed (Kesanakurti et al. [18]. Analysis of a 2 µL aliquot collected post-extraction from all samples yielded consistent Ct values ranging from 17 to 22, thereby validating the enrichment protocol prior to HTS library construction.

### 3.3. HTS Library Quantification

Libraries derived from the dsRNA enriched extracts were sequenced by HTS across seven independent runs, each comprising 48 libraries (Table 3). HTS libraries were quantified, yielding concentrations ranging from 0.2 to 67 nM, and were normalized prior to pooling and sequencing. Post-sequencing evaluation demonstrated that library concentration was a reliable predictor of method success. Libraries with concentrations between 0.0 and 0.4 nM exhibited a 94% failure rate (17/18 samples), whereas those within the 0.5–1.5 nM range showed a reduced failure rate of 27% (4/15 samples). All libraries with concentrations >1.5 nM yielded acceptable HTS results.

Based on these results, a concentration threshold of 1.5 nM was established for reliable sequencing, while libraries with concentrations <0.5 nM were excluded. Although failure rates increased with sample dilution (from 5.2% for undiluted samples to 17.5% at the 50-fold dilution), at least one replicate of each sample achieved a concentration ≥1.5 nM, suggesting that most library failures were stochastic rather than systematic.

### 3.4. HTS Data Quality Control Analysis

All seven sequencing runs met Illumina sequencing quality assurance criteria. Following sequencing and demultiplexing, Illumina FASTQ files were imported into Virtool and assessed using FastQC (v0.11.9). All samples satisfied the quality metrics for Quality Distribution at Read Position, Nucleotide Composition at Read Positions, and Read-wise quality. Three datasets, one replication each from VC05 (25-fold), VC08 (undiluted) and VC08 (10-fold), showed suboptimal profiles for Nucleotide Composition at Read Positions. However, because all expected viruses were successfully detected (see criteria below) in these datasets, they were retained for subsequent analysis.

### 3.5. Determining Criteria for Defining High-Quality Sample HTS Data

**Figure 1:**
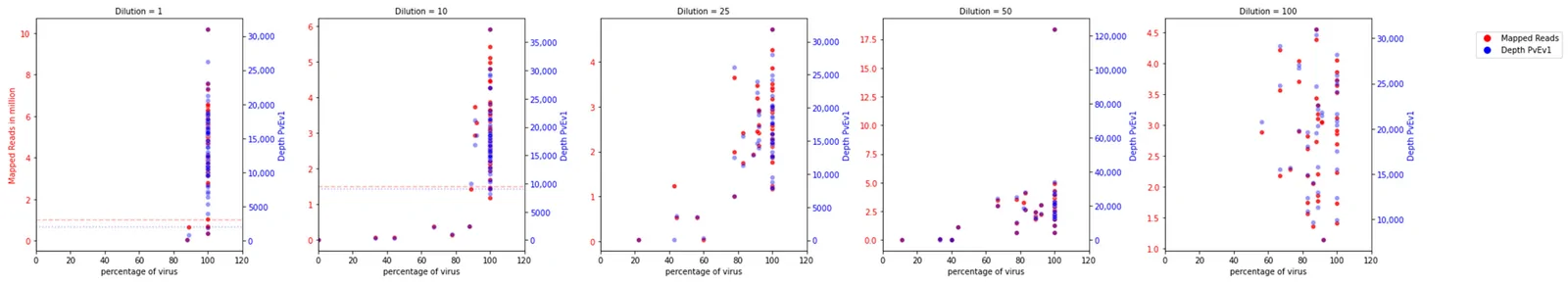
Relationship between mapped reads (MR), PvEV1 depth, and the percentage of expected viruses detected in individual HTS datasets across dilutions. Five scatter plots are presented: undiluted, 10-fold, 25-fold, 50-fold, and 100-fold dilution samples. The x-axis denotes the percentage of expected viruses detected in each HTS dataset. The primary y-axis (left) denotes MR (in millions), and the secondary y-axis (right) denotes PvEV1 depth. Individual data points represent HTS datasets with red for MR and blue for PvEV1 depth. Red dashed lines indicate thresholds for MR, and blue dashed lines indicate thresholds for depth of PvEV1 for undiluted and 10-fold dilution.

In addition to establishing criteria for defining a positive virus detection (see section below), HTS datasets were evaluated to determine general quality thresholds, ensuring sufficient sensitivity for virus detection when viruses were present. The total number of mapped reads (MR) to the virus database (in millions) and depth of reads to the spike-in control PvEV1 were plotted against the percentage of known viruses detected across the dilution series for each VC, yielding consistent trends (Figure 1).

Among 58 undiluted samples (undiluted VC01 to VC09 included replicates a/b/c/d/g/h (9 × 6) = 54; VC10 included only four replicates, a/b/c/d, as we did not have extra samples to run experiment 6 and 7; therefore, 54 + 4 = 58 samples), 55 datasets detected 100% of expected viruses. Of these, 53 had MR values between 1 and 10, while two had MR values < 1. The remaining three datasets achieved only 90% detection, with MR values < 1, and failed to detect grapevine virus H (GVH), grapevine rupestris vein feathering virus (GRVFV), and grapevine yellow speckle viroid 1 (GYSVd1). These three datasets were excluded from subsequent analysis. Accordingly, an MR-based threshold of ≥1 MR was established (Figure 1). PvEV1 depth values ranged from 43 to 31,117. The three datasets with incomplete virus detection had the lowest depths (43, 827, and 1062; Figure 1). Of the 55 HTS datasets with 100% virus detection, 54 had depth values > 1825. Accordingly, a PvEV1 depth-based threshold of ≥2000 was established (Figure 1).

For 10-fold-diluted samples, 48 datasets achieved 100% virus detection, five detected 80–90% and five < 80% (Figure 1). The 48 datasets with complete detection, together with four of the five with 90% detection, had MR values between 1.4 M and 6 M. GSyV1, GRVFV and GYSVd1 were inconsistently detected at this dilution. The remaining six datasets with lower detection rates had MR values of <1 M. Accordingly, an MR-based threshold of ≥1.5 M was established (Figure 1). Forty-six datasets with 100% virus detection had a PvEV1 depth > 9000 while two had slightly lower depths of 8231 and 8974. Four datasets failed to detect GSyV1 and GRVFV and had a PvEV1 depth > 9000. Accordingly, a PvEV1 depth-based threshold of ≥9000 was established (Figure 1). Another six datasets with 30–90% detection had PvEV1 depth values < 5000. These were the same ones with MR values below thresholds and were excluded from further analysis.

At the 25-fold and 50-fold dilutions, only 50% of the datasets detected all the expected viruses, indicating a substantial decline in detection sensitivity (Figure 1). At 100-fold dilution, variability increased markedly, and no reliable thresholds could be defined because relationships between MR values and detection rates were inconsistent (Figure 1). Despite this variability, a PvEV1 depth threshold of ≥9000 remained a useful indicator of sequencing library quality across diluted samples, consistent with observations from the 10-fold dilution datasets (Figure 1).

### 3.6. Determination of a Positive Virus Detection from the Sample HTS Data

Pathoscope outputs were used to summarize viral genomic coverage and weight across the HTS datasets. Coverage reflects the proportion of each viral genome, monopartite or multipartite, covered by sequencing reads, while weight represents the relative abundance of reads mapping to a virus species compared with all virus-associated reads detected. Analyses were performed using species-level, non-redundant coverage and weight values that combine all detected isolates within a species, providing enhanced sensitivity for virus detection. Coverage and weight values were recorded for all viruses expected in each VC sample and were treated as true positives. In addition, non-expected grapevine viruses detected due to cross-contamination during VC sample preparation, as well as grapevine-associated viruses, and non-target background viral reads inherent to the bioinformatic analysis, referred to as “noise”, were also observed. These detections were inconsistent across the VC set and could not be quantitatively defined during sample preparation; therefore, they were considered artefacts rather than true positives. Nonspecific virus detections were classified as true observations but not included as a positive detection. Together, coverage and weight metrics were used to evaluate virus presence across samples and to establish threshold limits for reliable detection.

#### 3.6.1. Genome Coverage

**Figure 2:**
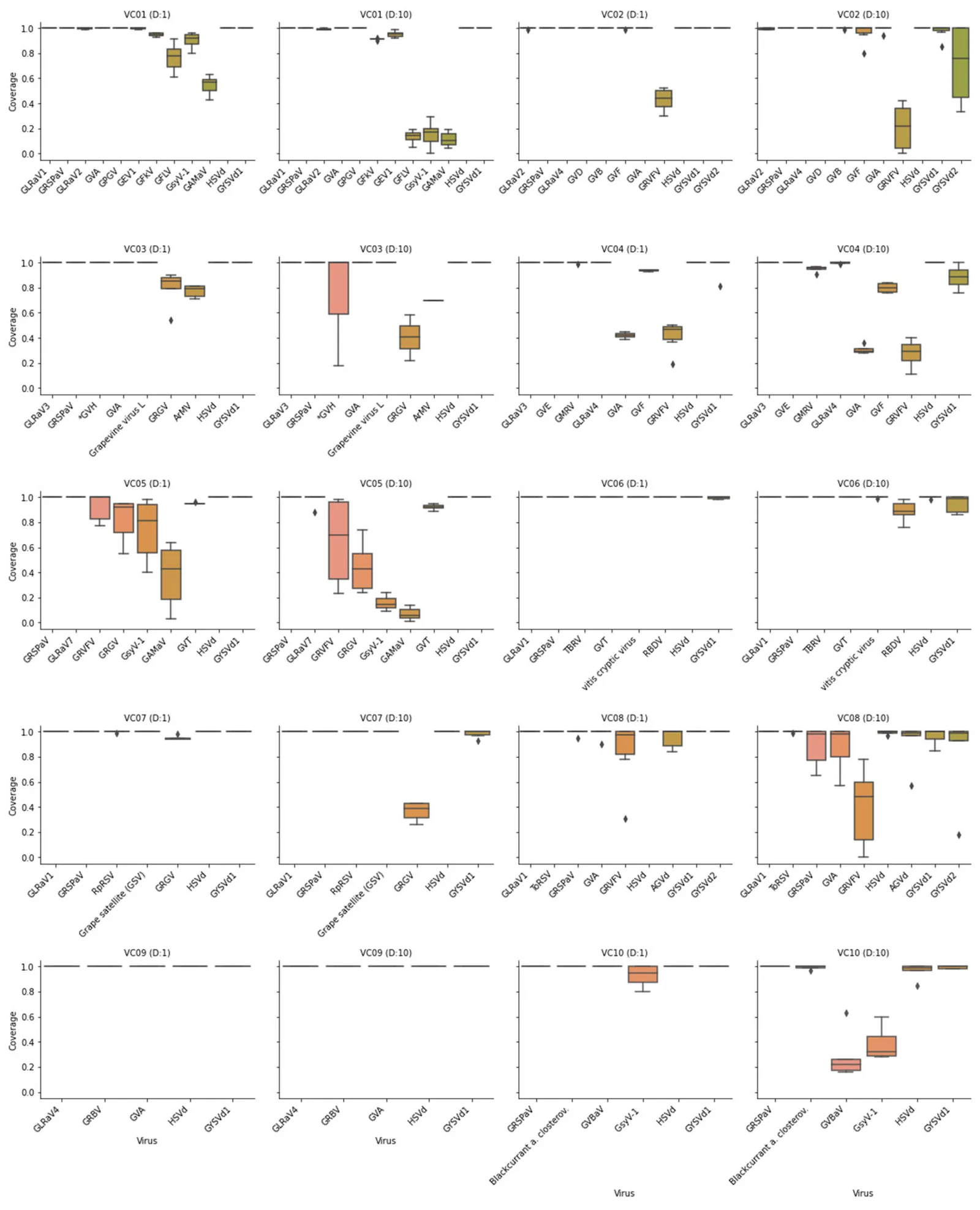
Distribution of viral genome coverage values in undiluted (D:1) and 10-fold-diluted (D:10) virus composite (VC) samples. Box plots for undiluted samples, the minimum replicate n = 4. For 10-fold-diluted samples, the minimum n = 3 for the HTS datasets. Panels correspond to VC01-VC10 arranged in five rows and four columns, with the viruses and viroids present in each VC shown on the x-axis. The y-axis shows genome coverage (0.0–1.0). GVH = (GVH/GVM specific coverage).

Figure 2 shows the genome coverage values and ranges for each expected virus and viroid detected in undiluted and 10-fold-diluted HTS datasets, based on 4–6 replicates per sample generated by two analysts across all 10 VC samples. Complete data for all dilution levels are provided in Supplementary Figure S1. A total of 85 expected virus and viroid occurrences are present across the 10 VC samples.

For most viruses in each HTS dataset, coverage is either 1.0 (complete genome) or close to 1.0 (near-complete genome), indicating sequencing saturation. As samples progress from undiluted to 10-fold dilution, coverage generally decreases as expected, particularly for viruses that exhibit less than complete coverage in the undiluted samples (Figure 2). A representative example is VC01, which shows notable reductions in coverage for grapevine fanleaf virus (GFLV), grapevine Syrah virus 1 (GSyV1), and grapevine asteroid mosaic-associated virus (GAMaV) when comparing undiluted samples to the 10-fold dilution (Figure 2). This trend is consistent across the 10 VC samples. Some viruses, exemplified by grapevine rupestris stem pitting-associated virus (GRSPaV), present in all VCs except VC04 and VC09, consistently exhibit complete or near-complete coverage in both undiluted and 10-fold-diluted samples. In contrast, several grapevine tymoviruses [GRVFV, GSyV1, GAMaV, and grapevine red globe virus (GRGV)] generally exhibited lower coverage values in the VC samples in which they were present (Figure 2). Overall, coverage for most viruses was near saturation in undiluted samples. Coverage declined with increasing dilution for a subset of viruses, with the largest decreases observed for those that exhibited incomplete coverage in undiluted samples (Supplementary Figure S1).

The viruses with lower coverage showed greater variability among replicates than those with higher coverage (Figure 2). For example, in VC01, six viruses, grapevine leafroll-associated virus 1 (GLRaV1), grapevine leafroll-associated virus 2 (GLRaV2), GRSPaV, grapevine virus A (GVA), grapevine pinot gris virus (GPGV), and grapevine enamovirus 1 (GEV1), as well as two viroids, hop stunt viroid (HSVd) and GYSVd1, exhibited coverage values of 1.0 (complete genome coverage), with little to no variation among replicates. In contrast, lower abundance viruses, based on the HTS results, including, grapevine fleck virus (GFkV), GFLV, GSyV1, and GAMaV exhibited lower coverage values and greater variability among replicates (Figure 2). This pattern was consistently observed across the 10 VC samples. Viruses with higher genome coverage generally exhibited little to no variability among replicates. In contrast, several grapevine tymoviruses (GRVFV, GSyV1, GAMaV, and GRGV) as well as GYSVd1, showed greater variability among replicates in the VC samples in which they were present (Figure 2). Furthermore, as dilution increased from undiluted to 10-fold dilution and higher, dilution levels and genome coverage generally decreased and became more variable, with some viruses showing substantial reductions in coverage. This trend is evident from the larger and downward-shifted box plots observed at higher dilution levels (Supplementary Figure S1).

**Figure 3:**
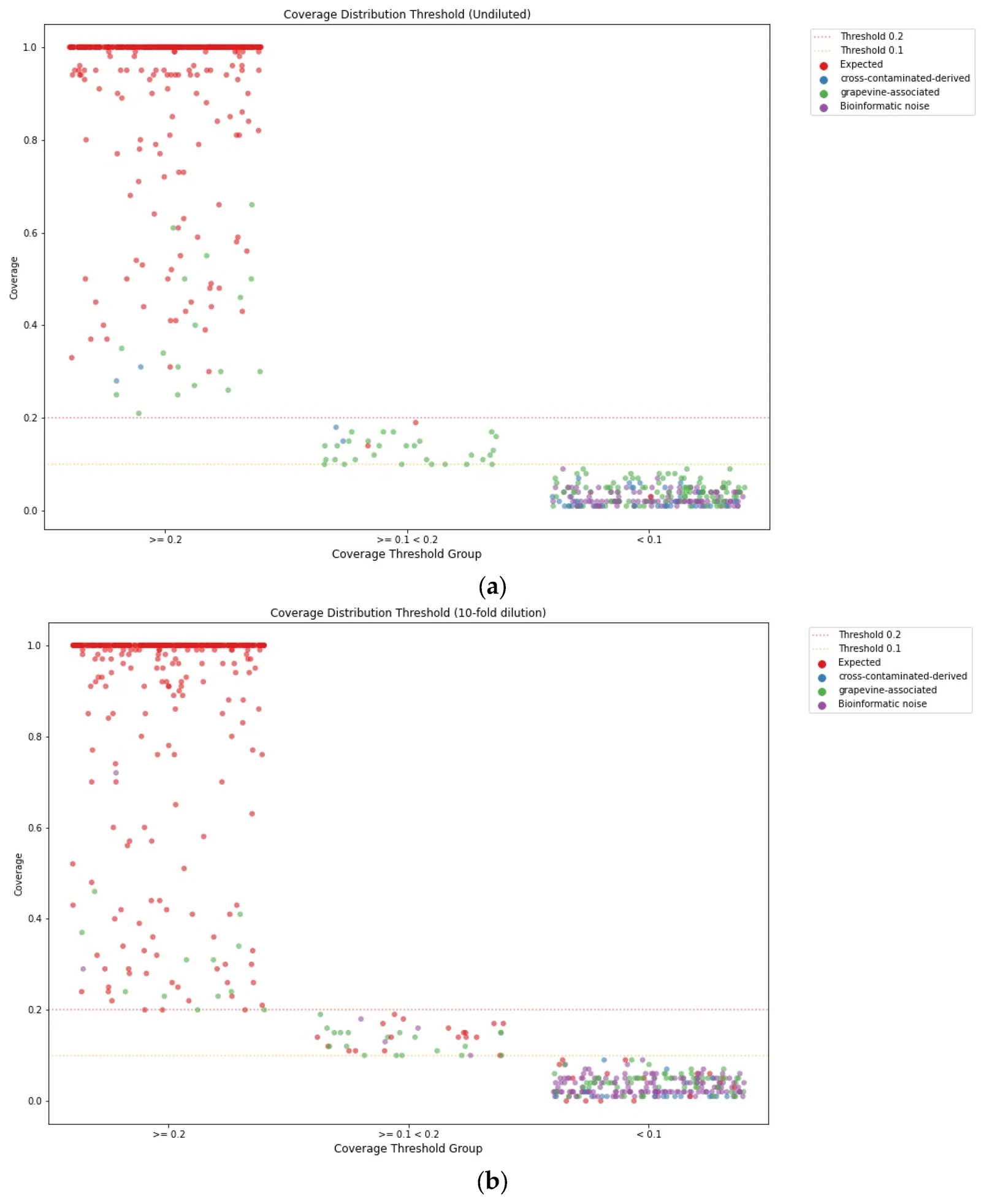
Distribution of viral genome coverage values in undiluted and 10-fold-diluted samples. Strip plots show genome coverage values for (**a**) undiluted and (**b**) 10-fold-diluted samples. The x-axis groups detections into three discrete coverage categories: (1) ≥0.2, (2) ≥0.1 < 0.2, and (3) <0.1. The y-axis shows the proportion of the viral genome covered, ranging from 0.0 (0%) to 1.0 (100%). Red and yellow dotted lines indicate the genomic coverage thresholds. Red points represent “expected” viruses. Blue, green, and purple points represent “non-expected” detections arising from cross-contamination, grapevine-associated viruses, and bioinformatic noise, respectively.

Coverage values for all the recorded virus in undiluted HTS datasets were plotted, revealing three distinct groups (Figure 3a). Each data point represents the coverage value of a virus detected across all replicates (4–6 per sample) from the 10 VC samples. Group 1 (≥0.2 coverage) included all true positives (see Section 3.7), with most values clustering near 0.8–1.0. This group also included cross-contamination-derived virus detections (GFLV, GSyV1) and grapevine-associated viruses (grapevine alphapartitivirus, grapevine fanleaf virus satellite RNA, WClMV, ClYMV, RCVMV). Group 2 (≥0.1 and <0.2 coverage) primarily included virus cross-contamination-derived virus detections (GLRaV-3, GFLV) and grapevine-associated viruses (grapevine alphapartitivirus, WCIMV, ClYVV, ClYMV, RCVMV, WCCV2). Group 3 (<0.1 coverage) consisted primarily of random bioinformatic noise, along with some cross-contamination-derived virus detections and grapevine-associated viral artefacts. Although viruses in this group were detected by the bioinformatic pipeline, they were considered artefacts rather than true-positive detections.

Among the expected viruses in undiluted samples, the vast majority were found in Group 1 (≥0.2 coverage). Only two viruses, GRVFV and GAMaV, were observed in Group 2 (≥0.1 and <0.2 coverage), and only one occurrence of GAMaV was observed in Group 3 (<0.1 coverage).

The trends observed in undiluted samples were largely maintained in the 10-fold dilution datasets (Figure 3b). Most expected viruses remained within Group 1 (≥0.2 coverage). However, 21 virus occurrences, representing GFLV, GAMaV, GSyV1, GRVFV, and GVBaV, were observed in Group 2, while 10 virus occurrences, all corresponding to GFLV, GAMaV, or GSyV1, fell within Group 3.

At higher dilution levels, establishing clear coverage-based thresholds became increasingly difficult because coverage values for low-titre viruses consistently declined below 0.2.

#### 3.6.2. Viral Weight

**Figure 4:**
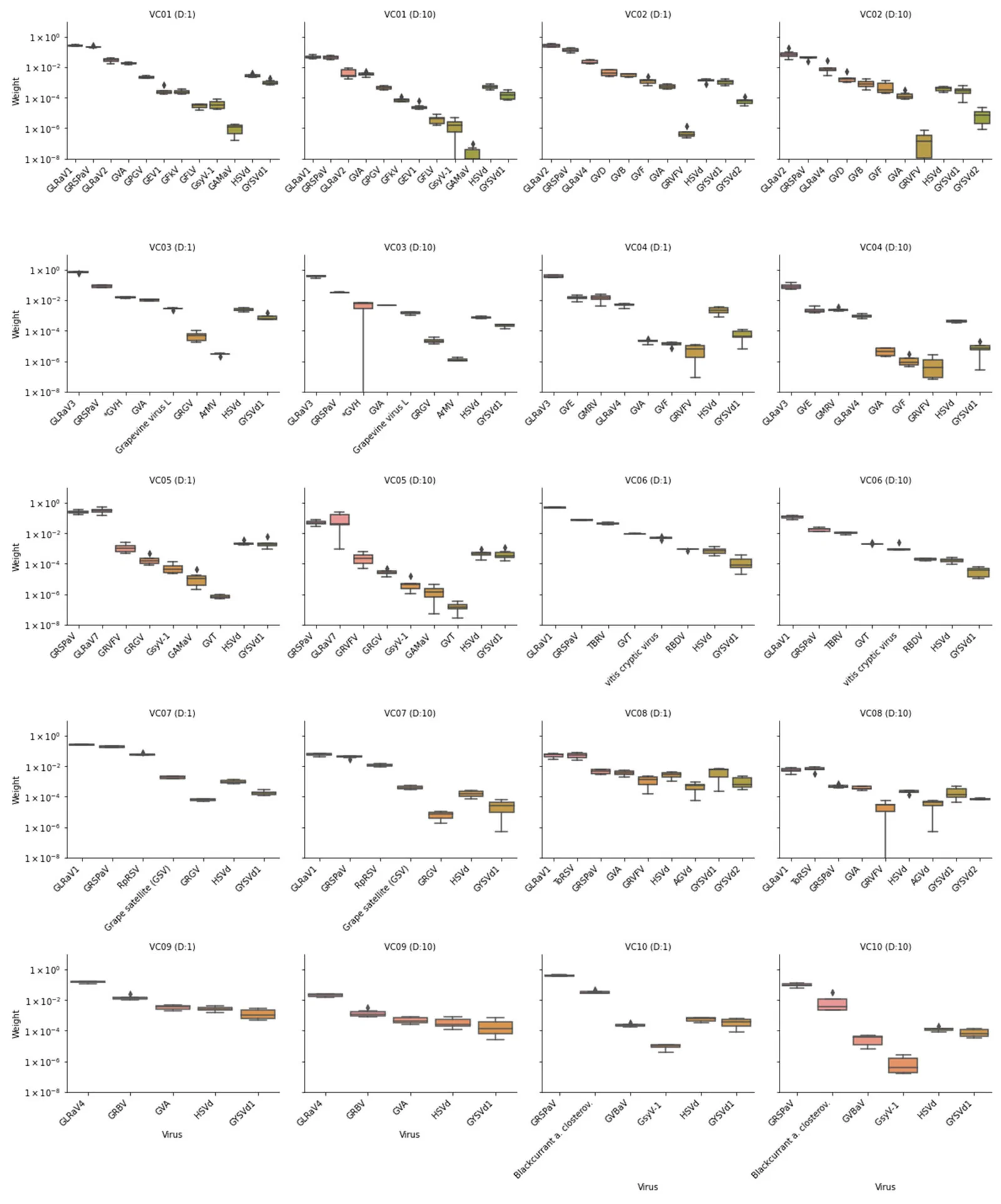
Distribution of viral weight values in undiluted (D:1) and 10-fold-diluted (D:10) virus composite (VC) samples. Boxplots for undiluted samples, with the minimum replicate n = 4. For 10-fold-diluted samples, the minimum n = 3 for the HTS datasets. Panels correspond to VC01–VC10 arranged in five rows and four columns, with the viruses and viroids present in each VC shown on the x-axis. The y-axis shows viral weight values on a logarithmic scale ranging from 1 × 10^1^ to 1 × 10^−8^. GVH = (GVH/GVM specific weight).

The proportion of reads mapping to each virus relative to the total number of reads mapping to all viruses within a sample was plotted for the undiluted and 1:10 dilution datasets (Figure 4). Supplementary Figure S2 presents the corresponding plots for all dilution levels. These plots do not include the internal spike-in control PvEV1; however, reads mapping to PvEV1 were included in the calculation of the total virus-associated reads. Consequently, the sum of all weight values for a given sample equals 1.0. Because the same amount of BBTS spike containing PvEV1 was added to each sample regardless of dilution, the relative weight of PvEV1 increased as sample dilution increased. The weight plots illustrate the broad dynamic range of viral abundance, spanning several orders of magnitude and reflecting the relative titre of each virus within a sample.

As observed for coverage, viruses with higher weight values exhibited greater consistency across VC samples, as indicated by the box plots, whereas viruses with lower weight values showed greater variability (Figure 4). For example, in VC01 (Figure 4), weight values for the 12 viruses and viroids ranged from a high of 3.16 × 10^−1^ (GLRaV1) to a low of 1.20 × 10^−6^ (GAMaV). Most viruses with higher weight values exhibited a narrow distribution indicating low variability among replicates. In contrast, GSyV1 and GAMaV showed a much greater vertical spread in their box plots, indicating higher variability in weight values among replicates. Finally, viroids were generally present at intermediate abundance (1 × 10^−3^ to 1 × 10^−5^) and exhibited moderate variability (Figure 4). The trend was observed across the 10 VC samples. Higher abundance viruses, exemplified by GRSPaV, GLRaV1, 2, and 3, exhibited little to no variation among replicates within sam ples. In contrast, several grapevine tymoviruses, including GRVFV, GSyV1, GAMaV, and GRGV, as well as grapevine yellow speckle viroids 1 and 2 (GYSVd1 and GYSVd2), exhibited greater variability among replicates in the VC samples in which they were present (Figure 4). This trend persisted across dilution levels. In undiluted samples, the majority of viruses exhibited little to no variability among replicates (Figure 4). As dilution increased, weight values generally decreased and became more variable, with some viruses showing substantial reductions in abundance. This pattern is evident from the larger and downward-shifted box plots observed at higher dilution levels (Supplementary Figure S2).

**Figure 5:**
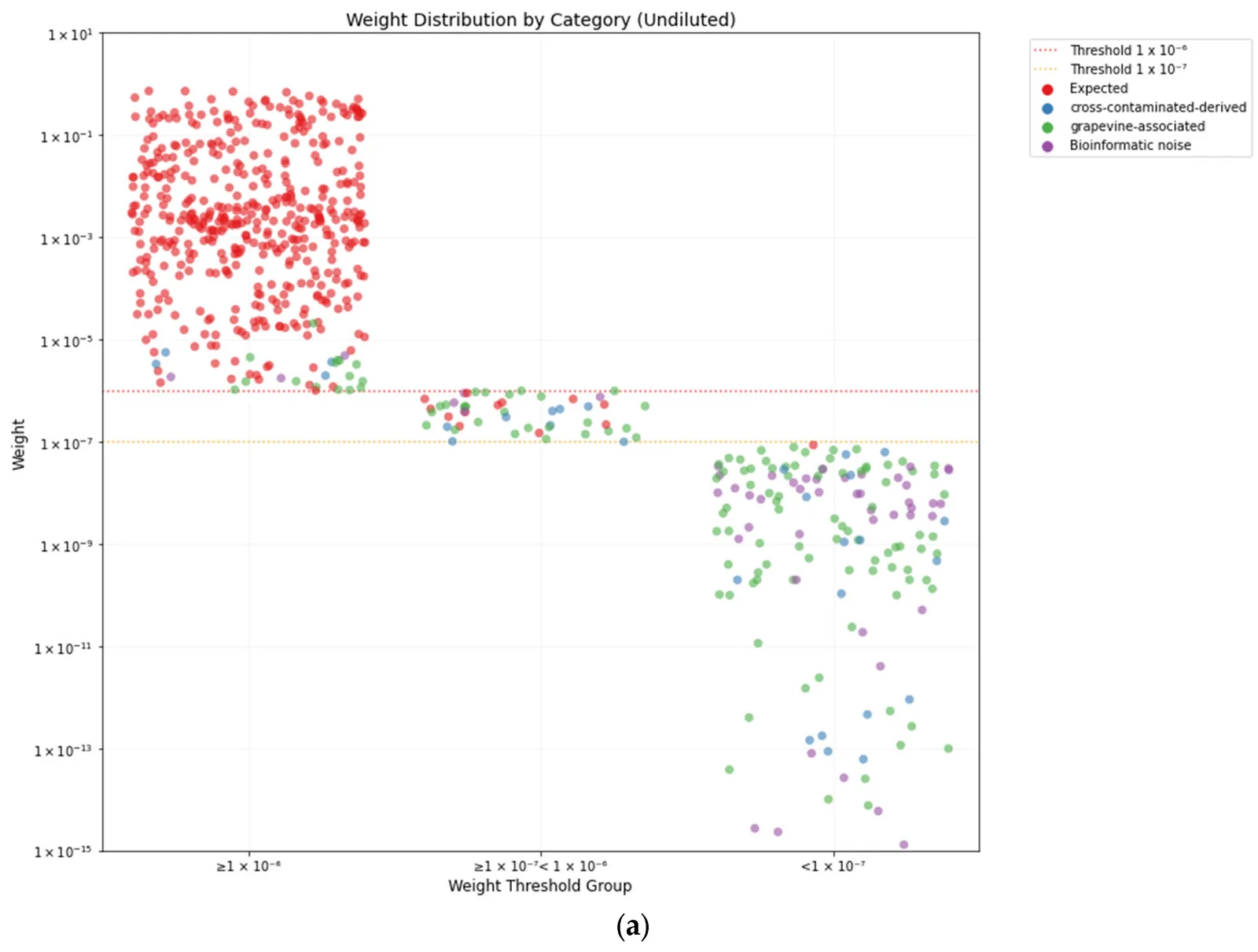

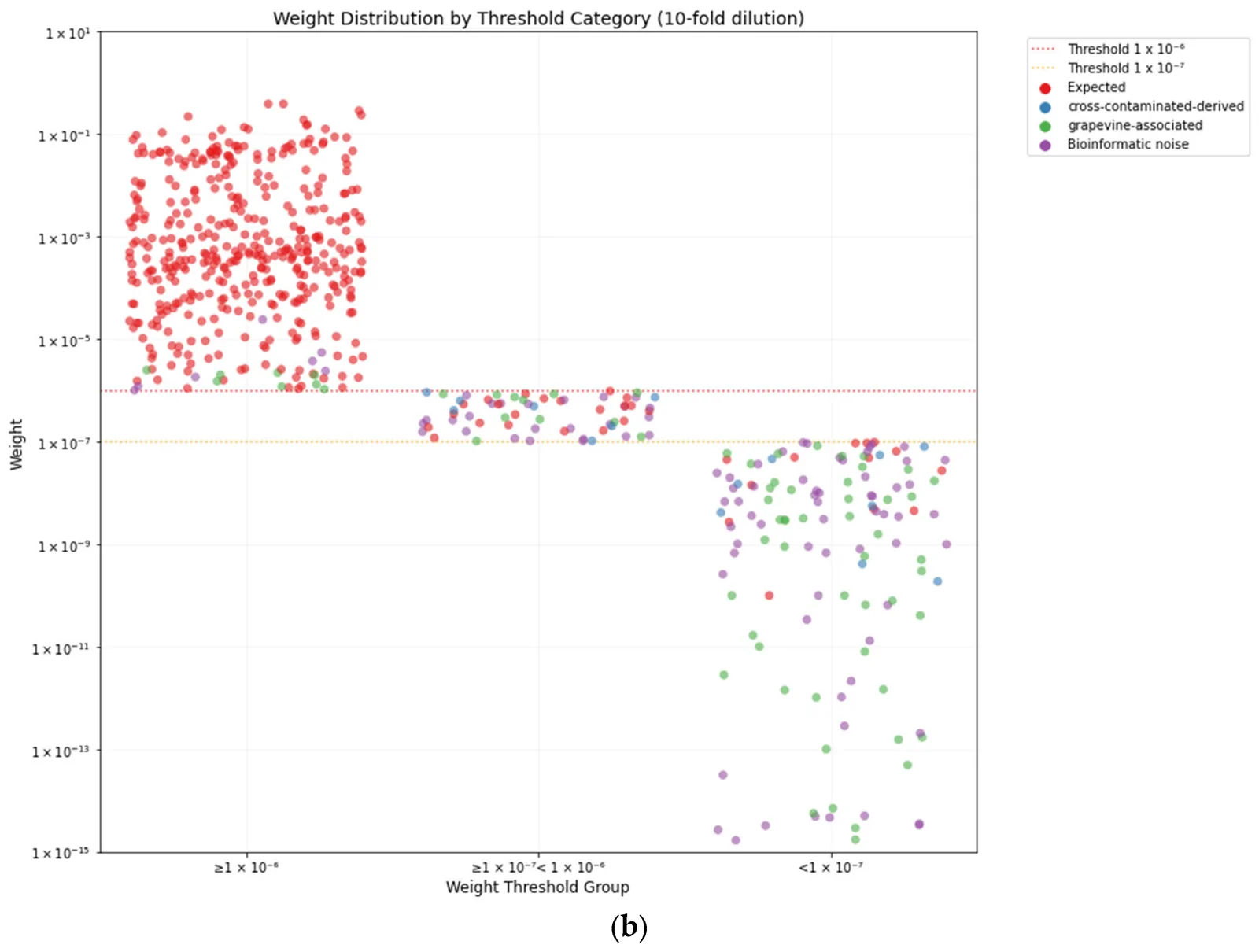
Distribution of viral weight values in undiluted and 10-fold-diluted samples. Strip plots show viral weight values for (**a**) undiluted and (**b**) 10-fold-diluted samples. The x-axis groups detections into three viral weight categories: (1) ≥1 × 10^−6^, (2) ≥1 × 10^−7^ < 1 × 10^−6^, and (3) <1 × 10^−7^. The y-axis represents viral weight values, ranging from 1 × 10^−15^ to 1 × 10^1^. Red and yellow dotted lines indicate viral weight thresholds. Red points represent expected viruses. Blue, green, and purple points represent non-expected virus detections arising from cross-contamination, grapevine-associated viruses, and bioinformatic noise, respectively.

Weight values for the recorded virus in all undiluted HTS sample data were plotted, revealing three distinct groups (Figure 5a). Group 1 (≥1 × 10^−6^) included all true-positive detections (see Section 3.7), as well as a small number of contaminating virus detections and grapevine-associated viruses. Group 2 (≥1 × 10^−7^ and <1 × 10^−6^) primarily comprised contaminating virus detections and grapevine-associated viruses. Group 3 (<1 × 10^−7^) consisted of virus detections that were considered artefacts rather than true positives. This group was composed primarily of bioinformatic noise, together with a small number of contamination-derived detections and grapevine-associated viral artefacts.

Among the expected viruses in undiluted samples (red dots), most were present in Group 1 (≥1 × 10^−6^). Twelve virus occurrences, all corresponding to GAMaV, GRVFV, or GVT, were present in Group 2 (≥1 × 10^−7^ and <1 × 10^−6^), while only a single occurrence of GRVFV was observed in Group 3 (<1 × 10^−7^). The trends observed in undiluted samples were largely maintained in the 10-fold dilution datasets (Figure 5b). Most expected viruses remained within Group 1. However, 21 virus and viroid occurrences, representing GSyV1, GRVFV, GVF, GAMaV, GYSVd1, GYSVd2, and AGVd, fell within Group 2, while 14 virus occurrences, corresponding to GRVFV, GAMaV, and GVT, were present in Group 3. At higher dilution levels, establishing clear weight-based thresholds became increasingly difficult because low-abundance viruses and viroids frequently fell below the proposed threshold values.

### 3.7. Detection/Contamination Threshold and Impact on Performance Criteria

Two approaches were used to establish thresholds for minimizing false-negative and false-positive virus detections: (1) integration of viral abundance (weight) and genome coverage metrics across the ten VC samples, and (2) characterization of false-positive detections through the comparison of expected virus detections with bioinformatic background noise.

#### 3.7.1. False Negatives

Viruses and viroids are unevenly distributed within grapevine tissues, and their abundance may vary among tissue types and across seasons, making sampling a critical factor influencing detection sensitivity. Improper sampling can result in false-negative detections, while cross-contamination during sample collection or processing remains a significant source of error. Consequently, the establishment of reliable detection thresholds is essential.

Using the ten VC sample, the impact of varying detection thresholds on the false-negative and false-positive rates was evaluated. In some cases, viruses fell into a higher category for one parameter (e.g., coverage) but in a lower or “questionable category for the other (e.g., weight), or vice versa.

For undiluted samples, two virus detections exhibited this discrepancy. For example, in VC05, GAMaV showed genome coverage < 0.1 (questionable), but weight > 1 × 10^−6^. Conversely, GRVFV showed weight < 1 × 10^−7^ (questionable), but genome coverage ≥0.1 < 0.2. In 10-fold-diluted samples, similar inconsistencies were observed for seven virus detections and one viroid detection across six samples. For example, in VC01, GSyV1 and GFLV exhibited genome coverage < 0.1 but weights ranging from ≥1 × 10^−7^ to 1 × 10^−6^, while GRVFV, GVT, and GYSVd2 showed weights < 1 × 10^−7^ but genome coverage ≥0.1 < 0.2.

Based on these observations, a conservative criterion for classifying a detection as false-negative was defined as weight < 1 × 10^−7^ and genome coverage < 0.1.

#### 3.7.2. False Positives

To evaluate false-positive detections, weight and genome coverage thresholds for expected viruses and viroids were compared with those of unexpected viral taxa detected within the same datasets. The same classification criteria were applied to all detections, identified through Pathoscope analysis (Figure 3 and Figure 5).

The following detections were excluded from further analysis: (1) grapevine-associated viruses/mycoviruses without confirmed infection (e.g., white clover mosaic virus, white clover cryptic virus 1 and grapevine alphapartitivirus), which have been reported in grapevine datasets but lack evidence of true infection [21]; (2) bioinformatic noise, consisting of detections with both weight and genome coverage below the “questionable” threshold (e.g., Hibiscus Singapore latent virus, Primula malacoides virus 1), indicative of weak or unreliable signals; and (3) cross-contamination-derived detections, where viruses from the expected HTS dataset were detected in unrelated samples with weight < 1 × 10^−7^ and genome coverage < 0.1, suggesting contamination rather than true infection.

#### 3.7.3. Threshold Definition and Decision Framework

Based on the integration of the parameters described above, a decision tree was developed to classify virus detection outcomes into three categories: positive (weight > 1 × 10^−6^ and genome coverage > 0.2), likely positive (weight between 1 × 10^−7^ and 1 × 10^−6^ and/or genome coverage between 0.1 and 0.2), or likely negative (weight < 1 × 10^−7^ and genome coverage < 0.1). Although this decision framework provides a standardized approach for interpreting HTS results, false-positive and false-negative classifications remain possible, particularly for low-titre viruses and samples affected by contamination or sampling variability. It should be noted that there is a complex relationship between weight and coverage that are influenced independently, depending on the number of viruses and viroids in a sample vs. their relative amount in the sample. Most of the time, the two values correlate with each other, however in rare cases they can separate. The amount of data generated from this study is insufficient to quantify this relationship. For diagnostic analysis, confirming results using another test method, such as PCR-based methods, is recommended.

### 3.8. Performance Evaluation of HTS Validation Metrics

Diagnostic accuracy relates to the ability of a test to discriminate between the presence (viral infection) and absence (healthy) of a target condition [22]. This discriminative potential can be quantified using a range of validation metrics. To comprehensively evaluate the diagnostic performance of the HTS method, six key metrics were assessed: accuracy, specificity, recall, false-positive rate (FPR), precision, and F1 score.

Overall, diagnostic performance declined progressively with increasing dilution. Accuracy was highest for undiluted samples (99.7%) and decreased to 91.4% at the 100-fold dilution, indicating that dilution negatively impacts overall detection performance. Specificity remained consistently high across all dilution levels, ranging from 99.4% in undiluted samples to 98% at 100-fold dilution. This minor decline suggests that dilution has limited impact on the ability to correctly identify true negatives, with an increase in false-positive detections. In contrast, recall decreased more substantially, from 100% in undiluted samples to 84.7% at 100-fold dilution, indicating reduced sensitivity and an increased likelihood of false-negatives at higher dilutions. The false-positive rate increased from 0.6% in undiluted samples to 2% at the 100-fold-dilution, further indicating a modest reduction in the method’s ability to correctly classify negative samples as dilution increased. Precision remained high across all dilutions, declining slightly from 99.4% to 97.6% indicating that the method maintained strong reliability in positive predictions producing few false-positives predictions despite increasing dilution. The F1 score, which balances precision and recall decreased from 99.7% for undiluted samples to 90.7% at 100-fold dilution. This trend reflects the growing imbalance between precision and recall as dilution increases, driven by a rise in false-negative detections, with a smaller contribution from false positives.

### 3.9. Repeatability

Repeatability is the degree of agreement observed when the same sample is analyzed under identical conditions, including the same method, operator, equipment, and a short time interval. Repeatability was assessed using both one-way and two-way intraclass correlation coefficient (ICC) models, which produced similar results. For the one-way ICC analysis, percent reads mapped (%RM) data from replicate analyses of 50 VC samples were evaluated separately for each analyst. The resulting ICC values were 0.84 and 0.89 for Analysts 1 and 2, respectively, indicating good repeatability. Values between 0.75 and 0.90 are considered good to excellent (ConductScience Bland–Altman Analyzer (v1.0.0)) and demonstrate a high level of repeatability for the HTS method across both analysts and evaluation metrics.

### 3.10. Intermediate Precision

Intermediate precision, also referred to as within-laboratory reproducibility, measures the variability observed when the same method is performed by different operators, on different days, within the same laboratory using the same equipment. Unlike repeatability, which assesses variation under identical conditions and by a single operator, intermediate precision captures operator-to-operator variability while keeping other factors relatively constant.

**Figure 6:**
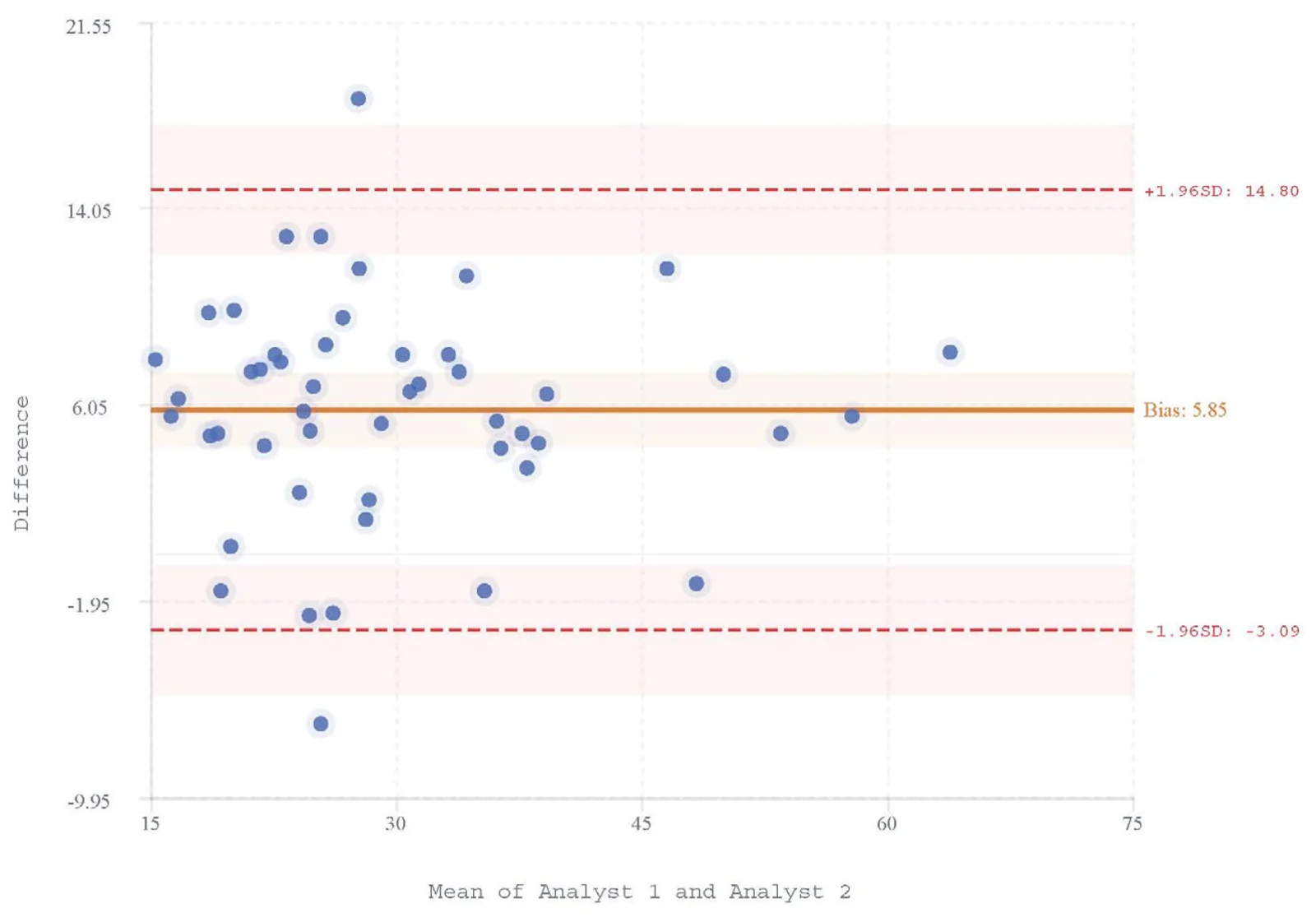
Bland–Altman plots assessing intermediate precision (within-laboratory reproducibility) between Analyst 1 and Analyst 2 based on percent reads mapped. Each point represents one paired measurement (n = 49). The solid orange horizontal line represents the mean difference between Analyst 1 and Analyst 2 of 5.85 (95% confidence level: 4.34 to 7.36), indicating a positive bias but no significant proportional bias (slope = 0.0029, *p* = 0.9614). The upper and lower red dashed lines represent the 95% limits of agreement, ranging from −3.09 to 14.80.

The mean difference in percent reads mapped (%RM) between Analyst 1 and Analyst 2 was 5.853 (95% CI: 4.3439 to 7.3623), indicating a positive bias. The 95% limits of agreement ranged from −3.0937 (CI: −5.7077 to −0.4797) to 14.7998 (CI: 12.1858 to 17.4138). No significant proportional bias was detected (slope = 0.0029, *p* = 0.9614). Sample size (n = 49) meets the minimum recommendation of 40 pairs (Figure 6). All three replicates failed in one sample, a 10-fold dilution of VC03 analyzed by Analyst 2. Consequently, the data for this sample were excluded from the analysis for both analysts.

### 3.11. Assessment of Background Contamination Using an Internal Control

Contamination can arise during both wet laboratory and bioinformatic processes. Three main sources were identified: (1) cross-contamination during sample preparation, whereby samples processed together occasionally exhibited consistent contamination across replicates; viruses attributed to such cross-contamination were excluded from further analysis; (2) technical errors during extraction, library preparation, or sequencing, which contributed to false-positive detections; and (3) bioinformatic errors during data analysis, which contributed to both false-positive and false-negative detections.

To quantify background contamination, a dedicated experiment was conducted using an internal control consisting of 48 wells: 45 healthy samples and three containing infected samples (two TBRV positive samples, and one VC07 sample). Among the 48 wells, eight exhibited low-level contamination with TBRV, with a maximum relative abundance of 1.7 × 10^−3^% and a median of 1.5 × 10^−5^%. TBRV positive samples are also infected with VCV, so VCV was also measured in this analysis. Two wells are contaminated with VCV, with a maximum relative abundance of 3.4 × 10^−5^% and the median relative abundance of 1.9 × 10^−5^%. Importantly, no wells exceeded the established detection thresholds, indicating that all observed signals fell within the background contamination range. Overall, these findings establish a quantifiable baseline level of background contamination for each virus across all wells. This baseline may assist in the interpretation of low-level detections.

## 4. Discussion

High-throughput sequencing (HTS) technologies have transformed plant virus diagnostics by enabling more sensitive and faster detection of viruses and viroids in routine plant health testing [8,12,23]. The HTS diagnostic workflow, consisting of tissue sampling, nucleic acid extraction, library preparation, sequencing, and bioinformatic analysis provides a comprehensive non-targeted platform for detecting diverse pathogens in a single assay. However, HTS validation approaches differ among laboratories depending on plant hosts, workflow-specific particulars, threshold criteria, and their consecutive technical and biological factors. This study is intended to serve as a practical example of how an HTS-based diagnostic test method can be systematically validated for plant virus and viroid diagnostics. Using an in-house developed HTS workflow for grapevine viruses and viroids representing multiple genome types, we describe a comprehensive validation strategy and assess key performance characteristics relevant to diagnostic testing. While the validation results are specific to the workflow examined, the study highlights general principles, methodological considerations, and critical factors that influence HTS diagnostic performance. As such, it provides a framework that can assist plant health laboratories, researchers, and industry stakeholders in developing, evaluating, and adapting validation approaches for their own HTS-based diagnostic systems.

A central finding of this work is the importance of rigorous quality control across all stages of the HTS workflow. While standard sequencing metrics (e.g., Phred scores, read counts, and nucleotide composition) were consistently met, they were insufficient alone for diagnostic interpretation. Additional criteria were required to ensure that sequencing data were suitable for reliable virus detection. The integration of internal controls proved essential for validation. Consistent Ct values for the BBTS spike-in control confirmed uniform dsRNA enrichment across all samples, and although not observed in this study, delayed Ct values would provide an early indicator of enrichment failure. Library concentration was identified as a key determinant of sequencing and diagnostic success. A clear transition in performance was observed, with libraries above 1.5 nM consistently yielding reliable results and those below 0.5 nM showing high failure rates. Importantly, dilution had a pronounced effect on both library quality and virus detectability.

The combined use of mapped reads (MR) and internal control depth represents an important advancement toward standardizing HTS-based diagnostics. Mapped reads integrate total sequencing output and the proportion of reads aligning to viral genomes, while internal control depth provides an independent measure of sequencing efficiency and consistency. Together, these metrics effectively predict whether sufficient sequencing information is present to detect viruses if they are present. Given the current lack of standardized quality criteria for HTS in plant virus diagnostics, the thresholds established in this study provide a practical and transferable framework for routine implementation. Collectively, mapped reads and PvEV1 depth provided robust and complementary indicators of sequencing adequacy, enabling the establishment of minimum thresholds required for confident virus detection. Samples meeting these criteria achieved near-complete detection (100% in undiluted samples and >99% at 10-fold dilution), demonstrating the effectiveness of these parameters in defining high-quality datasets (Figure 1). Undiluted samples contain higher concentrations of target viral nucleic acids and required lower sequencing depth thresholds to achieve complete detection. Based on observed performance, three MR-based quality categories were defined: good (≥1 MR; the red dashed line, Figure 1), questionable (0.5–1 MR), and poor (<0.5 MR). And a PvEV1 depth threshold of ≥2000 was defined as Good. In contrast, 10-fold-diluted samples required substantially higher thresholds for MR and PvEV1 depth to maintain sensitivity by minimizing false negatives. Based on the performance, three MR-based quality categories were defined: good (≥1.5 MR; the red dashed line, Figure 1), questionable (0.5–1.5 MR), and poor (<0.5 MR). PvEV1-based quality thresholds were established as: good (≥9000), questionable (2000–9000), and poor (<2000) (Figure 1). More libraries fell below the detection threshold in 10-fold dilutions resulting in loss of sensitivity in viral detection.

The progressive loss of sensitivity with increasing dilution helps define the practical limits of HTS-based detection. As target nucleic acid concentrations decrease, fewer viral genome fragments are represented in the sequencing data, resulting in reduced genome coverage and an increased likelihood of incomplete or missed detections. This behaviour is consistent with stochastic sampling effects expected at low template concentrations, where random variation in the number of viral molecules entering the workflow becomes increasingly important. Consequently, some viruses approached or fell below the detection threshold at higher dilutions, leading to an increased risk of false-negative results.

This effect was most apparent for a subset of viruses that consistently occurred at low relative abundance, including several members of the family *Tymoviridae* such as GAMaV, GRVFV, and GSyV1. In contrast, more abundant viruses, including GLRaVs and GRSPaV, maintained high genome coverage and consistent detection across dilution levels. These observations illustrate that diagnostic sensitivity is influenced not only by the number of sequenced reads but also by the biological characteristics and abundance of individual viruses within the sample.

The coverage and weight analyses provided complementary insights into detection reliability. Viruses with high abundance typically exhibited stable coverage and little variation among replicates, whereas detections near the limit of sensitivity showed greater variability and reduced genome recovery. The positive relationship between viral weight and genome coverage further supports the presence of a biologically meaningful detection threshold. Below a weight of approximately 1.0 × 10^−6^, genome coverage was generally low and inconsistent, while above this value coverage increased rapidly and frequently approached completeness. This pattern was observed across both undiluted and diluted samples.

These findings support the use of a multi-parameter interpretation framework rather than reliance on a single metric. By combining abundance (weight) and genome coverage, the three-tier classification system developed in this study provides a practical approach for distinguishing strong detections from weak or ambiguous signals. The framework recognizes that detections close to the analytical limit of the method require more cautious interpretation, while maintaining confidence in clearly positive and clearly negative results.

In addition to the expected target detections, several non-expected viral signatures were identified within the VC dataset, including grapevine viruses not anticipated in the healthy control material, grapevine-associated viruses, and low-abundance viral sequences attributable to background signals inherent to high-throughput sequencing and downstream bioinformatic analyses. Collectively, these signals were interpreted as analytical artefacts rather than true biological infections of the VC sample starting material. These detections were characterized by low read abundance, lack of consistent genome coverage, and poor reproducibility across replicate samples and control sets. True-positive detections in controlled HTS experiments are expected to demonstrate consistent recovery across replicates and concordance with known sample composition; however, the observed non-target viral signals occurred sporadically and were not reproducible across the VC dataset. The presence of non-expected grapevine viruses is consistent with low-level cross-contamination during sample handling and library preparation. Similarly, the detection of mycoviruses/grapevine-associated viruses likely reflects viruses associated with plant tissues, including endophytic or epiphytic fungi, rather than infection of the host plant relevant to the testing objective. Additionally, a subset of detections is attributable to bioinformatic noise, including nonspecific read mapping to related viral taxa, alignment to conserved genomic regions, and limitations of reference databases. These signals typically manifest as a small number of non-overlapping reads and do not meet commonly accepted thresholds for confident virus identification. Importantly, these non-target signals could not be quantitatively predicted, controlled, or consistently reproduced during sample preparation, and no correlation was observed between their presence and experimental variables. Taken together, the sporadic occurrence, low abundance, lack of reproducibility, and absence of biological relevance support the interpretation that these detections represent background analytical artefacts rather than genuine viral presence in the VC sample material.

High-abundance viruses (e.g., most GLRaVs, GRSPaV) consistently achieved near-complete genome coverage with low variability across replicates. In contrast, low-abundance viruses exhibited higher variability across replicates. This underscores the importance of internal controls such as PvEV1 for standardizing sequencing performance and ensuring sufficient depth for detecting low-abundance targets. The robustness of the method was further demonstrated through validation metrics and reproducibility assessments. High levels of accuracy, specificity, and precision across dilution series confirm strong diagnostic performance, while the observed decline in recall with increasing dilution reflects the expected sensitivity limitations at low viral concentrations. Repeatability and intermediate precision analyses revealed no significant operator-dependent bias, supporting the consistency of the method across analysts. Additionally, contamination analyses showed that background signals remained well below detection thresholds, although low-level contamination and cross-sample transfer highlight the importance of stringent laboratory practices and careful result interpretation.

Despite these strengths, some limitations remain. Specific wet-lab technical variables decisively influence HTS diagnostic outcomes. Despite the implementation of stringent contamination control measures, HTS detected trace levels of sample contamination, primarily associated with minor aerosolization in undiluted and low-dilution samples during plant tissue homogenization preparation. Although these detections were technically positive, they were classified as inconsistent contaminants based on their low frequency and were excluded from the true-positive dataset used for subsequent analyses. Virus-specific and biological factors also played a critical role in detection outcomes. Viral concentration can vary widely depending on plant genotype, physiological stage, and viral replication dynamics, limiting the generalizability of detection thresholds across all conditions. Of the 102 known grapevine viruses [24]. Several (e.g., GAMaV, GFkV, GSyV1, GRVFV, GRGV) are known to establish latent infections and often occur at low abundance in the HTS data regardless of sample and grapevine accession number suggesting their true presence is low, making them inherently more difficult to detect. In addition, the experimental removal of larger veins—where many phloem-limited viruses are concentrated, created a “worst-case” sampling scenario that likely reduced detection sensitivity compared to typical diagnostic samples. These findings emphasize that sampling strategy remains a critical determinant of diagnostic success and must be carefully optimized for certification and surveillance applications. Furthermore, the accuracy of viral detection is highly dependent on the completeness and quality of the reference genome library/database, which is critical for distinguishing closely related viruses. Limitations in this library as used by Pathoscope may have contributed to misclassification of GVH as GVM. In this case, an isolate identified as GVM (accession number MK492703) shares 67% identity to GVH, resulting in cross-detection between these two closely related viruses in a few diluted replicates of VC03. Therefore, the weight and coverage values of GVM were treated as those of GVH. These findings reinforce that HTS, while highly sensitive, is still subject to both technical and biological constraints.

In conclusion, this study establishes and validates a quantitative framework for HTS-based detection of grapevine viruses and viroids. By integrating sequencing quality metrics, internal controls, and combined genome coverage and weight thresholds, the workflow provides a robust, reproducible, and transparent approach for interpreting diagnostic HTS results. Although challenges remain for low-abundance viruses and highly diluted samples, the method demonstrated strong overall performance and clearly defined its operational limits. While this study focuses on the extraction of viral dsRNA, the validation methodology provided should be transferable to various other viral nucleic acid extraction types including libraries generated from total RNA and ribodepleted RNA.

Beyond the specific validation outcomes obtained for this in-house grapevine virus and viroid HTS workflow, this study serves as a practical example of how HTS-based diagnostic methods can be systematically validated for plant virus and viroid detection. Using a pathogen panel representing diverse genome organizations and biological characteristics, we describe a comprehensive validation strategy and evaluate key performance parameters relevant to diagnostic testing. While the specific performance metrics and detection thresholds reported here are workflow-dependent, the study highlights general principles, methodological considerations, and validation approaches that can inform the development, assessment, and implementation of HTS-based diagnostics across a broad range of plant health applications.

## Figures and Tables

**Table 1:** Virus and viroid content of the ten virus composite (VC) samples: Ten reference virus composite samples (VC01–VC10) selected for the validation study. The virus and viroids status of the plants were verified via previous RT-PCR and HTS.

Virus Comp.	Plants Used	Virus/Viroid	# of Viruses
VC01	3	GAMaV, GLRaV1, GLRaV2, GRSPaV *, GPGV, GVA, GEV-1, GFkV, GFLV, GsyV-1, HSVd *, GYSVd1	12
VC02	1	GLRaV2, GLRaV4, GRSPaV, GRVFV, GVA, GVB, GVD, GVF, HSVd, GYSVd1, GYSVd2	11
VC03	2	ArMV, GLRaV3, GRGV, GRSPaV *, GVA, GVH, GVL, HSVd *, GYSVd1 *	9
VC04	1	GLRaV3, GLRaV4, GMRV, GRVFV, GVA, GVE, GVF, HSVd, GYSVd1	9
VC05	2	GAMaV, GLRaV7, GRGV, GRSPaV *, GRVFV, GSyV-1, GVT, HSVd *, GYSVd1 *	9
VC06	2	GLRaV1, GRSPaV, GVT, RBDV, TBRV, VCV, HSVd, GYSVd1	8
VC07	2	GLRaV1, GRSPaV, GSV, RpRSV, GRGV, HSVd, GYSVd1	7
VC08	2	GLRaV1, GRSPaV, GRVFV, GVA, ToRSV, HSVd, GYSVd1, GYSVd2, AGVd	9
VC09	1	GLRaV4, GRBV, GVA, HSVd, GYSVd1	5
VC10 ^1^	2	BCCV-1, GRSPaV, GVBaV, GSyV-1, HSVd, GYSVd1	6
HC	1		0
		Total	85

Grapevine leafroll-associated virus 1 (GLRaV1), grapevine leafroll-associated virus 2 (GLRaV2), grapevine leafroll-associated virus 3 (GLRaV3), grapevine leafroll-associated virus 4 (GLRaV4), grapevine leafroll-associated virus 7 (GLRaV7), grapevine rupestris stem pitting-associated virus (GRSPaV), grapevine pinot gris virus (GPGV), grapevine virus A (GVA), grapevine virus B (GVB), grapevine virus D (GVD), grapevine virus E (GVE), grapevine virus F (GVF), grapevine virus H (GVH), grapevine virus L (GVL), grapevine virus T (GVT), grapevine enamovirus 1 (GEV-1), grapevine Syrah virus 1 (GSyV-1), grapevine asteroid mosaic-associated virus (GAMaV), grapevine rupestris vein feathering virus (GRVFV), grapevine fleck virus (GFkV), grapevine red globe virus (GRGV), grapevine fanleaf virus (GFLV), arabis mosaic virus (ArMV), tomato black ring virus (TBRV), raspberry ringspot virus (RpRSV), tomato ringspot virus (ToRSV), raspberry bushy dwarf virus (RBDV), grape satellite virus (GSV), blackcurrant closterovirus 1 (BCCV-1), grapevine muscat rose virus (GMRV), vitis cryptic virus (VCV), grapevine red blotch virus (GRBV), gooseberry vein banding-associated virus (GVBaV), hop stunt viroid (HSVd), grapevine yellow speckle viroid 1 (GYSVd1), grapevine yellow speckle viroid 2 (GYSVd2), Australian grapevine viroid (AGVd), and healthy control (HC). * Indicates viruses in all plants used to make up the composite. ^1^ Indicates that one of the plants is a *Ribes* plant.

**Table 2:** The detailed list of viruses and viroids: Thirty-four viruses and four viroids are included in this validation study. Taxonomic nomenclature follows the latest International Committee on Taxonomy of Viruses (ICTV) classification. Official genus and species names are italicized in accordance with standard virology nomenclature. Virus genome nucleic acids (N.A.) include positive-sense single-stranded RNA [ssRNA (+)], negative-sense single-stranded RNA [ssRNA (−)], double-stranded RNA (dsRNA), single-stranded DNA (ssDNA), double-stranded DNA (dsDNA), and single-stranded circular RNA (sscRNA). Genome organization segments (Seg.) range from non-segmented to tripartite genomes. Full virus and viroid names corresponding to the acronyms used in the text are provided in this table.

Genus	Species	Virus Name	Abrev.	N.A.	Seg.
*Ampelovirus*	*Ampelovirus univitis*	grapevine leafroll-associated virus 1	GLRaV1	ssRNA (+)	Non
*Ampelovirus*	*Ampelovirus trivitis*	grapevine leafroll-associated virus 3	GLRaV3	ssRNA (+)	Non
*Ampelovirus*	*Ampelovirus tetravitis*	grapevine leafroll-associated virus 4	GLRaV4	ssRNA (+)	Non
*Closterovirus*	*Closterovirus vitis*	grapevine leafroll-associated virus 2	GLRaV2	ssRNA (+)	Non
*Velarivirus*	*Velarivirus septemvitis*	grapevine leafroll-associated virus 7	GLRaV7	ssRNA (+)	Non
*Foveavirus*	*Foveavirus rupestris*	grapevine rupestris stem pitting-associated virus	GRSPaV	ssRNA (+)	Non
*Trichovirus*	*Trichovirus pinovitis*	grapevine pinot gris virus	GPGV	ssRNA (+)	Non
*Vitivirus*	*Vitivirus alphavitis*	grapevine virus A	GVA	ssRNA (+)	Non
*Vitivirus*	*Vitivirus betavitis*	grapevine virus B	GVB	ssRNA (+)	Non
*Vitivirus*	*Vitivirus deltavitis*	grapevine virus D	GVD	ssRNA (+)	Non
*Vitivirus*	*Vitivirus epsilonvitis*	grapevine virus E	GVE	ssRNA (+)	Non
*Vitivirus*	*Vitivirus phivitis*	grapevine virus F	GVF	ssRNA (+)	Non
*Vitivirus*	*Vitivirus etavitis*	grapevine virus H	GVH	ssRNA (+)	Non
*Vitivirus*	*Vitivirus lambdavitis*	grapevine virus L	GVL	ssRNA (+)	Non
*Vitivirus*	*Vitivirus muviti*	grapevine virus M	GVM	ssRNA (+)	Non
*Foveavirus*	*Foveavirus tafvitis*	grapevine virus T	GVT	ssRNA (+)	Non
*Enamovirus*	*Enamovirus GEV*	grapevine enamovirus 1	GEV1	ssRNA (+)	Non
*Marafivirus*	*Marafivirus syrahense*	grapevine Syrah virus 1	GSyV-1	ssRNA (+)	Non
*Marafivirus*	*Marafivirus asteroides*	grapevine asteroid mosaic-associated virus	GAMaV	ssRNA (+)	Non
*Marafivirus*	NA	grapevine rupestris vein feathering virus	GRVFV	ssRNA (+)	Non
*Maculavirus*	*Maculavirus vitis*	grapevine fleck virus	GFkV	ssRNA (+)	Non
*Maculavirus*	NA	grapevine red globe virus	GRGV	ssRNA (+)	Non
*Nepovirus*	*Nepovirus foliumflabelli*	grapevine fanleaf virus	GFLV	ssRNA (+)	Bi
*Nepovirus*	*Nepovirus arabis*	Arabis mosaic virus	ArMV	ssRNA (+)	Bi
*Nepovirus*	*Nepovirus nigranuli*	tomato black ring virus	TBRV	ssRNA (+)	Bi
*Nepovirus*	*Nepovirus rubi*	raspberry ringspot virus	RpRSV	ssRNA (+)	Bi
*Nepovirus*	*Nepovirus lycopersici*	tomato ringspot virus	ToRSV	ssRNA (+)	Bi
*Idaeovirus*	*Idaeovirus rubi*	raspberry bushy dwarf virus	RBDV	ssRNA (+)	Bi
*Virtovirus*	NA	grape satellite virus	GSV	ssRNA (+)	Bi
*Closterovirus*	*Closterovirus uniribi*	blackcurrant closterovirus 1	BCCV-1	ssRNA (+)	Non
*Rubodvirus*	*Rubodvirus argentinaense*	grapevine muscat rose virus	GMRV	ssRNA (−)	Tri
*deltapartitivirus*	NA	Vitis cryptic virus	VCV	dsRNA	Bi
*Grablovirus*	*Grablovirus vitis*	grapevine red blotch virus	GRBV	ssDNA	Non
*Badnavirus*	*Badnavirus venaribis*	gooseberry vein banding-associated virus	GVBaV	dsDNA	Non
*Hostuviroid*	*Hostuviroid impedihumuli*	hop stunt viroid	HSVd	sscRNA	Non
*Apscaviroid*	*Apscaviroid alphaflavivitis*	grapevine yellow speckle viroid 1	GYSVd1	sscRNA	Non
*Apscaviroid*	NA	grapevine yellow speckle viroid 2	GYSVd2	sscRNA	Non
*Apscaviroid*	*Apscaviroid austravitis*	Australian grapevine viroid	AGVd	sscRNA	Non

**Table 3:** Overview of validation experiments: Summary of the seven validation experiments, including the virus composite (VC) samples analyzed, the number of replicates, and the analyst responsible for each experiment.

Experiment	VC Samples	Replicates	Analyst
1	VC02, VC03, VC04, VC05	a, b	1
3	VC02, VC03, VC04, VC05	c, d	2
2	VC06, VC08, VC09, VC10	a, b	1
4	VC06, VC08, VC09, VC10	c, d	2
5	Healthy only samples, TBRV, VC07	^2^ H, TBR, VC07	1
6	VC01, VC07; VC02–VC06 ^1^; VC08–VC10 ^1^	a, b, g	1
7	VC01, VC07; VC02–VC06 ^1^; VC08–VC10 ^1^	c, d, h	2

^1^ In experiments 6 and 7, the run includes only undiluted and 10-fold dilution only for the VC02, VC03, VC04, VC05, VC06, VC08, VC09 and VC10. ^2^ 45 healthy (H) replicates, 2 TBRV positive replicates, 1 VC07 replicate.

## Data Availability

The data that support the findings of this study are available from the corresponding author upon reasonable request.

## Supplementary Materials

The following supporting information can be downloaded at https://www.mdpi.com/article/10.3390/pathogens15090998/s1, Supplementary Document S1: HTS Test Method for Plant Viruses and Viroids. Supplementary Document S2: dsRNA Enrichment for HTS Using the Hamilton Nimbus Presto. Supplementary Document S3: HTS Library Preparation from dsRNA Using the Hamilton MICROLAB^®^ STAR™. Figure S1: Distribution of viral genome coverage values in all dilutions for virus composite (VC) samples. Figure S2: Distribution of viral weight values in all dilutions for virus composite (VC) samples. Note that the values given Supplemental Document S1, which is an operational SOP differ slightly from the value thresholds presented in this study and will eventually be updated. What is more significant is how value thresholds are determined by a diagnostic laboratory when developing their own SOP.
