# Supplementary material for: A Validation Study of a High-Throughput Sequencing Workflow for the Detection of Grapevine Viruses and Viroids
Source: Pathogens. 2026 Sep 21;15(9):998. doi: 10.3390/pathogens15090998 (PMC13610249; doi:10.3390/pathogens15090998)
Supplement: Supplementary file 1 [file pathogens-15-00998-s001.zip › Supplemental Document S1. HTS TEST METHOD FOR PLANT VIRUSES AND VIROIDS.pdf]

Supplemental Document S1: HTS TEST METHOD FOR PLANT VIRUSES AND VIROIDS

1. INTRODUCTION

High Throughput Sequencing (HTS) technology is a highly sensitive diagnostic approach that enables the simultaneous screening of a single sample against hundreds of known plant viruses. In addition to detecting established pathogens, the sequence data generated through HTS can also reveal the presence of new or previously uncharacterized viruses or variants. Its exceptional sensitivity and broad scope make HTS a powerful alternative for primary virus screening, offering advantages over traditional diagnostic methods, such as ELISA, RT-PCR, and field or greenhouse bioassays.

Figure S3. Plant virus diagnostics using an HTS workflow

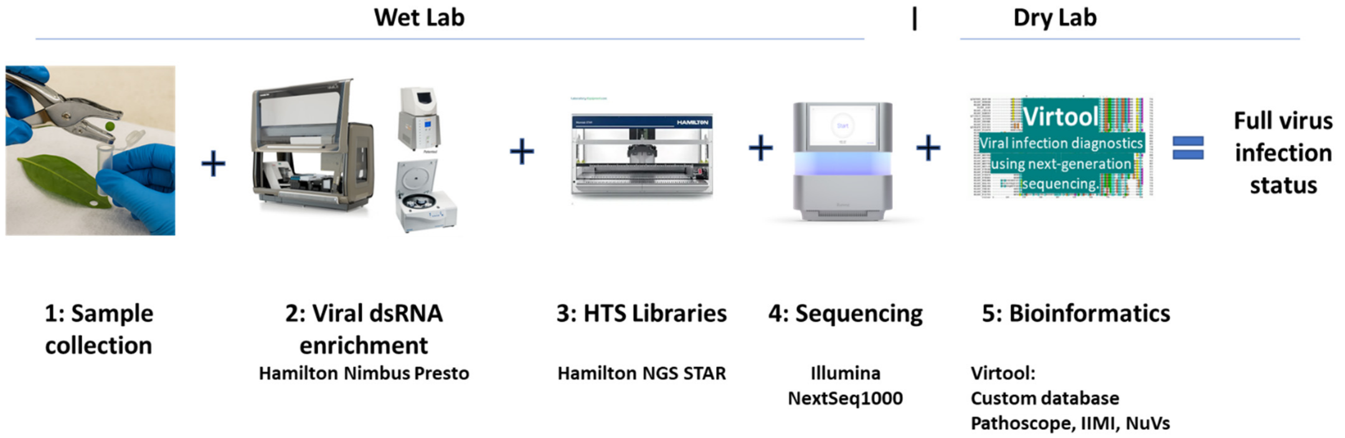

The High Throughput Sequencing (HTS) workflow follows a series of well-defined stages: (1) sample collection and preparation, (2) enrichment of viral double-stranded RNA, (3) library preparation, (4) sequencing, and (5) data analysis and interpretation.

1.1 Double Stranded RNA:

Most plant viruses possess RNA genomes. Detection of these viral genomes in infected plant material using HTS is achieved through the extraction and sequencing of double-stranded RNA (dsRNA). Because plants do not typically produce high molecular weight dsRNA, this molecule serves as a natural and highly specific target for plant virus diagnostics. All plant DNA viruses and viroids tested are also detected from enriched dsRNA extracts. However, it is possible that some DNA viruses will not be detectable for dsRNA enriched extracts.

To evaluate the quality of the dsRNA enriched extracts, library preparation, and sequencing workflow, a small amount of internal control is added to each sample prior to extraction. This control consists of two endornaviruses, *Phaseolus vulgaris* endornavirus 1 and 2 (PvEV-1/-2), naturally present in the cultivar Black Bean Turtle Soup (BBTS). A real-time PCR assay targeting PvEV-1 has been developed to verify the success of dsRNA extraction.

1.2 High Throughput Sequencing Library Construction:

The process of creating a HTS library begins with the fragmentation of the enriched dsRNA into smaller pieces. These fragments are converted into complementary DNA (cDNA) using reverse transcriptase and random primers, with strand specificity ensured through specialized reagents. The cDNA fragments are then modified by adding an adenine base, ligated to sequencing adapters, and purified. Finally, the library is enriched through PCR amplification, producing a high-quality cDNA library ready for sequencing.

1.3 Sequencing:
This test method is validated on the Illumina NextSeq1000, though other platforms may be used if they meet minimum requirements for read length, total reads, and quality. Using the XLEAP P2 100 cycle kit, the NextSeq1000 generates ~40 Gb of data, 400 million reads. In this method single-ended 100 base read lengths are used. Paired end, 2 x 50 base reads are an option that still needs to be evaluated. A sequencing run requires three components: flow cell, RSB Tween 20 reagent, and reagent cartridge. Each component contains an embedded chip with a serial number and expiry date. The NextSeq1000 software performs system checks before sequencing and provides built in quality assurance monitoring to evaluate run performance.

1.4 Data Handling and Analysis:
HTS sample data management and analysis are accomplished using Virtool (https://www.virtool.ca/) . Virtool and its dependencies are created and updated in Github. (https://github.com/virtool). Two workflows can be used to determine presence of a virus in a sample, Pathoscope and NuVs. Both Pathoscope requires a plant virus genome reference database.

1.4.1 Plant Virus Genomic Database and Host Genomic Database
The "Plant Virus" Database, hosted on GitHub, serves as the primary reference resource for Virtool. While Virtool supports the creation of specialized databases, the Plant Virus Database plays a central role in plant virus diagnostics. Through scheduled updates, it scans the latest version of GenBank for new isolates and species, ensuring consistency with current virus nomenclature. Each updated release is uploaded to Virtool with a new version number. Although the process is largely automated, manual curation remains critical to correct errors in GenBank accessions, which are tracked across successive versions. Some entries also require expert judgment, particularly those labeled as "host plant–associated viruses," many of which are not true plant viruses; these questionable records are maintained separately in a dedicated file. To improve efficiency and reduce computational costs, the database is collapsed at a 99% identity threshold for all isolates within a species.
Both Pathoscope and NuVs rely on host genome sequence data for subtraction. The selection of host genomes can be updated as needed, and if a specific host genome is unavailable for the sample under analysis, the taxonomically closest known host genome should be used instead.

1.4.2 Pathoscope
Is the original Virtool workflow for detecting known viruses in a sample. It uses a read-mapping approach, aligning each read from the HTS library against a reference database of plant virus sequences and applying statistical analysis to the results. A key limitation is that Pathoscope can only identify viruses represented in the database. Detection accuracy also declines when sequence divergence between database isolates and the HTS sample exceeds tolerable thresholds, and the degree of genomic variability differs widely among virus species. For this reason, it is important to regularly update the Plant Virus Database for new species and isolates.

1.4.3 *NuVs*
This workflow is designed to detect novel viruses or divergent isolates of known viruses. For an HTS workflow to serve as an alternative to a bioassay, analysis cannot rely solely on Pathoscope; evaluation with NuVs is also required. NuVs assembles contigs from unmapped reads generated by Pathoscope, translates them, and screens the resulting protein products for motifs characteristic of plant viruses using Hidden Markov Models.

2. PREPARATION OF THE TEST

2.1 Collection and Preparation of Samples

2.1.1 Sample Collection

2.1.1.1 It is essential that all samples collected be labelled appropriately.

2.1.1.2 Collect a negative (healthy) control representing one of the species being tested.

2.1.1.3 Aim to collect at least one positive control for a range of pathogens to be tested. A composite positive control for multiple viruses can be used. At least one positive control should be of the same species and physiological stage as the samples being tested.

Note: It is recognized that positive and negative controls of the same species and physiological stage are not always available. If tissue of the same physiological stage cannot be obtained from an infected plant of the same species, use what positive tissue is available. If an infected plant of the same species is not available, use tissue from an infected plant of a different species, a lyophilized positive control obtained from a commercial source, or an isolate of the virus that is being maintained in a herbaceous host. Consult the responsible Section/ Program Head before using one of these alternative positive controls. If tissue of the same physiological stage cannot be obtained from a healthy plant of the same species, use a closely related species. If a closely related species is not available, consult the responsible Section/ Program Head.

2.1.1.4 Collect, label and store negative and positive controls in a similar fashion to the samples. When collected at the same time, collect and handle in order of likelihood of infection:

Samples under test – assumed negative
Heat therapy tips
Samples under test – known/ suspected positive
Positive controls
Healthy controls

2.1.1.5 **Gloves must be worn at all times when collecting foliar/blossom tissue.** This includes when collecting tissue from negative or healthy samples, from samples of unknown disease status, and from positive, or suspected positive, control material. **Change gloves between plants** (or between samples if a composite sample is collected).

Note: It is essential to harvest samples from different areas of the plant as some viruses are unevenly distributed.

2.1.1.6 Collect samples according to the table below

Tissue type	Collection	Storage
Cambium	The number of cuttings harvested depends on size. Generally, 2-3 cuttings, 30 cm to 45 cm long, are harvested from different scaffolds of the plant. Cuttings are collected while dormant, which means once the wood has hardened off to bud break.	Bind cuttings together, label, and store in plastic bags at 4°C ± 3°C for up to one month (*Vitis spp.*) or up to three months (tree fruit).
Leaves	Collect two to four leaves from a one-gallon potted<br>plant, more if the plant is larger, from different areas of the test plant. Leaves may be placed directly into<br>labelled grinding bags.	Store leaves for up to one week at 4°C ± 3°C, or longer term at ≤- 60°C (refer to 4.1.1.9)

2.1.1.7 Place sample bags and/or tubes to be stored long term at ≤ - 60°C into a larger plastic bag or tube.

Note: In some instances, the virus titre in plant material may drop below detectable limits when subjected to freezing.

2.1.2 Sample Preparation

2.1.2.1 Retrieve stored samples and keep at 4°C ± 3°C until ready to process. Remove only the number of samples required for processing at one time to prevent drying out.

2.1.2.2 Gloves must be worn at all times when processing samples. This includes when processing tissue from negative or healthy samples, from samples of unknown disease status, and from positive, or suspected positive, control material. **Change gloves between samples**.

2.1.2.3 Prepare samples according to the table below:

Tissue type	Sample Preparation	Measurement and storage
Cambium	Perform a visual inspection of the dormant wood by scratching the bark. The cambial layer must be green for the sample to be considered viable. The cambial layer is located directly beneath the bark. Use a knife or similar tool to harvest cambial tissue from the cuttings by creating very fine shavings. Long shavings should be cut into smaller pieces Decontaminate the knife, or similar tool,<br>before use and between samples by wiping/spraying blade with 3% hydrogen peroxide, or use a disposable single-use blade for each sample. Collect the scrapings for each sample on a clean sheet of paper or paper towel and ensure the work area is free from debris.<br>Take care to include as little bark as possible.	Combine the scrapings from each stick in the sample, mix well, and transfer approximately 0.4 g to .6 g to a labelled 5 ml grinding tube. Keep the unprocessed wood at 4°C ± 3°C for possible further testing. The remainder of the scrapings may be kept and stored up to three days at 4°C ±3°C or at ≤ -60°C if longer term storage is required (refer to 4.1.1.9).<br><br>To all samples collected in 5 ml grinding tube one disc of BBTS control leaf material can be added at this stage.
Leaves	If several leaves are collected, sub sample by coring or tearing a small but equal portion from the basal end of each leaf. Ensure a portion of the petiole and mid-vein region of each leaf is sampled (see Figure S3)	Transfer approx. 0.4 g to 0.6 g to a labelled 5 ml grinding tube. Foliage, whether intact or sub-sampled, can be stored for up to one week at 4°C ± 3°C, or at ≤- 60°C if longer term storage is required (refer to<br>4.1.1.9).<br><br>To all samples collected in 5 ml grinding tube one disc of BBTS control leaf material can be added at this stage.
Lyophilized (freeze-dried) tissue	Freeze Dried Tissue: Freeze dried (lyophilized) material is not used for routine diagnostic testing. If a freeze-dried sample is required as a control, consult with the appropriate Section/ Program Head for instruction on dilution.	

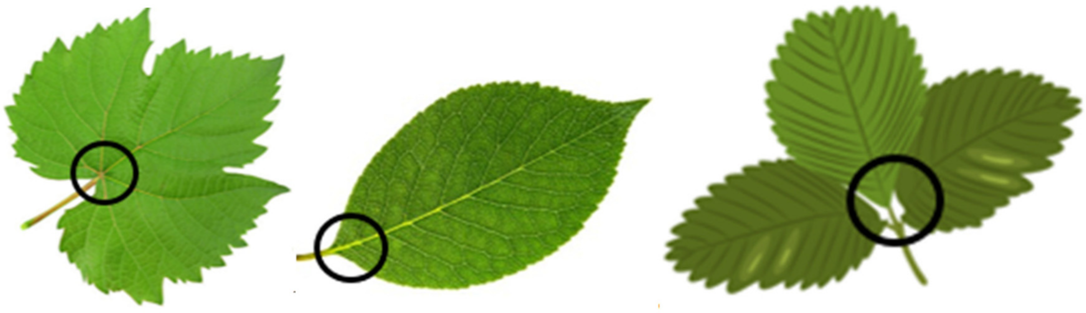

Compound Leaf (e.g. Fragaria spp.) | Simple leaf with lobes (e.g. Vitis or Ribes spp.) | Simple leaf without lobes (e.g. Malus, Prunus, Pyrus spp.)

Figure S4. Target sampling area, including portions of the midrib and petiole, for various leaf shapes.

2.1.2.4 Refer to dsRNA Extraction for HTS Using the Hamiton Nimbus Presto for preparing the plant material. BBTS extraction control (prepared according to Text S1) must be added to the tubes.

3. PERFORMANCE OF THE TEST

3.1 Final Preparation of Samples

3.1.1 Samples should now be in 5ml extraction tubes. If the samples have been stored, verify that they are still suitable for testing, for instance, there is no decaying or browning of tissue. If samples are not suitable, collect new sample or if they are still in plastic bags, transfer to 5 ml extraction tubes. If a BBTS control disc has not been added to each 5 ml extraction tube, this is to be completed now.

3.1.2 **DsRNA Enrichment:** Refer to dsRNA Extraction for HTS Using the Hamiton Nimbus Presto.

3.1.3 Record **all** the samples, including the controls.

3.1.4 **Strongly Recommended:** dsRNA enrichments are checked by real-time PCR for the BBTS control. Refer to Test Protocol CPHGD1301. Typical Ct values are between 17-22. Later Ct values may indicate a problem with the dsRNA enrichment. This step can be optional unless quantification of the HTS library in 6.2 is poor, or a new host plant species is being tested.

3.2 HTS Libraries preparation: Hamilton MICROLAB® STAR™

3.2.1 Once 48 dsRNA extractions have been completed which includes the healthy and positive controls, proceed with preparing the library for each sample according to
HTS Library Preparation from dsRNA Using the Hamilton MICROLAB® ™

3.2.2 Enter the total number of samples to be tested into the worksheet, including positive and negative controls, in the appropriate cell, noting the sample order or plate sample layout, in the order in which they will be set-up in the library reaction.

3.2.3 Proceed with library preparation as per HTS Library Preparation from dsRNA Using the Hamilton MICROLAB® ™

3.3 Library quantification and normalization

3.3.1 Follow instructions for qubit quantification and normalization in SOP L035 – HTS Library Preparation dsRNA

3.3.2 Performance criteria for HTS Libraries

- Qubit values of 0.2 or less have a 100% failure rate
- Qubit values of 0.4 or less have more than a 90% failure rate
- Qubit values between 0.5 and 1.5 have a failure rate of less than a 10%
- Qubit values greater than 1.5 have less than a 1% failure rate

3.3.3 If the Qubit values are less than 0.2 or the library is otherwise considered unsuccessful, repeat the library using a new aliquot from the same dsRNA extracts. If the Qubit values is still 0.2 or less, extract can be checked using the QPCR (see 3.1.4)

3.4 Sequencing: NextSeq1000

Performance criteria for Sequencing

- Target: ≥ than 90% of bases at Q30 or higher.
- Target: ≥ 60% cluster occupancy and quality through % Clusters Passing Filter (PF)
- Target: total reads output for a P2 1 x 100 flow cell is approximately 400 million reads with a total data output of approx. 40Gb

Text S1. Preparing *Phaseolus vulgaris* cultivar Black (Bean) Turtle Soup (BBTS)

Phaseolus vulgaris cultivar Black (Bean) Turtle Soup (BBTS) is naturally infected with the dsRNA *Phaseolus vulgaris* endornavirus -1 (PvEV1). This makes it an ideal internal control for dsRNA enrichment methods, HTS libraries and sequencing.

Original source BBTS, Veseys Seeds Ltd, cat #61040.

Seeds should be rejuvenated at least once every 5 years. BBTS plants are grown as in the protocol below and allowed to go to seed. Plants used for collecting seed are to be checked for the presence of PvEV1 using the by real-time PCR assay. All plant should be positive for PvEV1 before seed collection.

Protocol:

1. From seed, grow BBTS plants in a controlled environment at 21 ± 3 ˚C with 14h or light.
2. Leaves can be harvested after 1-8 weeks
3. Using a 50 mm^2 leaf punch, generate the required number of leaf discs.

4. Place each disc in individually capped strip tubes.
5. Leaves can be stored at −70 ˚C.

6. Alternatively, place each disc in 96 well microtitre plate.
7. Freeze dry the discs in the microtire plate.
8. Seal plate with Microseal F (or other adhesive, pierceable cover suitable for cold storage).
9. Store at −70 ˚C for longer term storage.

10. Use one disc per dsRNA extraction.
