# Supplementary material for: A Validation Study of a High-Throughput Sequencing Workflow for the Detection of Grapevine Viruses and Viroids
Source: Pathogens. 2026 Sep 21;15(9):998. doi: 10.3390/pathogens15090998 (PMC13610249; doi:10.3390/pathogens15090998)
Supplement: Supplementary file 1 [file pathogens-15-00998-s001.zip › Supplemental Document S2. Nimbus Presto dsRNA Extraction Method.pdf]

Supplemental Document S2: Nimbus Presto dsRNA Extraction Method

Preparation of the Nimbus Reagents

1.1 Calculate and prepare fresh reagent mixes (Binding Mix, Turbo Master Mix, and Rebinding Mix).

1.2 Required volume needed includes; reaction volume + dead volume. To calculate necessary reagents; Divide Required volume / Per reaction volume = # of equivalent reactions. Round up to the whole number and add 5 reactions to give the Final reaction #. Multiple all reagents by Final reaction #. *Each master mix will have a different Final reaction #.

1.3 Binding Mix

	Required / Per reaction		
	______ mL / 0.37mL =		x ______
10X STE		95 ul	______
100% ETOH		175 ul	______
Promega Resin (vortex 1min immediately before dispensing)		100 ul	______
Reaction volume		370 ul	

1.4 Turbo Master Mix

	Required / Per reaction		
	______ mL / 0.2mL =		x ______
10X Turbo Dnase buffer		20 ul	______
Nuclease free H2O		177 ul	______
Turbo Dnase @ 2U/ul - 6U total		3 ul	______
Rnase T1 @1000U/ul - 100U total		0.1 ul	______
Reaction volume		200 ul	

1.5 Rebinding Mix

	Required / Per reaction		
	______ mL / 0.1mL =		x ______
0.5M EDTA		9 ul	______
10X STE		30 ul	______
100% ETOH		61 ul	______
Reaction volume		100 ul	

Sample Preparation for Extraction

1.6 Subsample leaf or dormant wood tissue and store until needed at -80ºC. Add BBTS spike-in.

To a 5 mL standing tubes containing a single 8mm stainless steel bead, add;

350-550 mg fresh weight leaf or dormant wood tissue

100-250 mg freeze dried leaf tissue

If necessary, spin down samples briefly prior to storage.

1.7 Remove sample tubes from –80 ºC and let tubes warm at room temperature for 2 mins. Add 3.5 mL Hybrid Extraction Buffer A to samples.

1.8 Place in GenoGrinder2 010 with appropriate adapter block and grind for 1 min at 1750 rpm.

1.9 Vortex tubes for 5 sec. Sample should appear well ground. If not, invert the tube a few times to dislodge material and repeat the grinding step once. Cambium samples will require an additional grinding step: Centrifuge for 3 min to bring plant material to the bottom of the tube. Repeat grinding as in Step 8.11 for cambium and as needed for fresh and freeze-dried. **Note: the BBTS control disk should be fully ground.**

1.10 Centrifuge samples to produce cleared plant lysates for the Sample Plate.

For 5 mL standing tubes, use Yingtai TD5A (or equivalent) with swinging buckets and spin at 4000 rpm (2,630 x g) for 15 mins. Ensure that all buckets are properly balanced.

1.11 5 mL sample tubes, containing pelleted debris and cleared plant lysate, will be added to the sample tube carrier as indicated by the sample layout template.

Preparation of the Nimbus Presto

1.12 From the Input parameters window, input the number of samples to be run. For Sample volume – 600 µL, Elution volume – 110 µL. For 'Sample Input Labware' - 0, indicate the automated sample transfer from 5 mL tubes to the Sample plate.

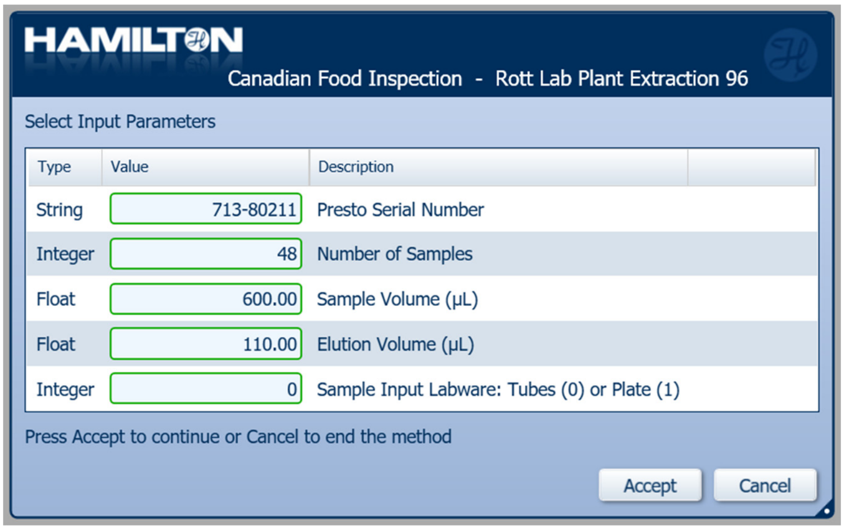

1.13 Transfer the indicated volumes for wash buffers STE-18 and ST-80 to 200 mL reservoir tubs. Add tubs to the appropriate positions of the carrier. Press OK.

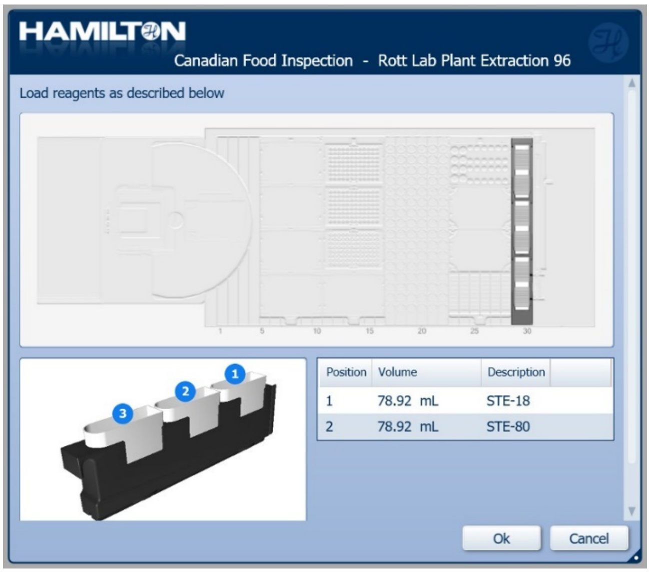

1.14 Required volumes for reagent mixes are displayed. Load the Elution Buffer (10 mM Tris pH 8) as well as freshly prepared Binding Mix, Turbo Master Mix and Rebinding Mix to labelled 60 mL reservoir tubs and add to the carrier positions indicated.

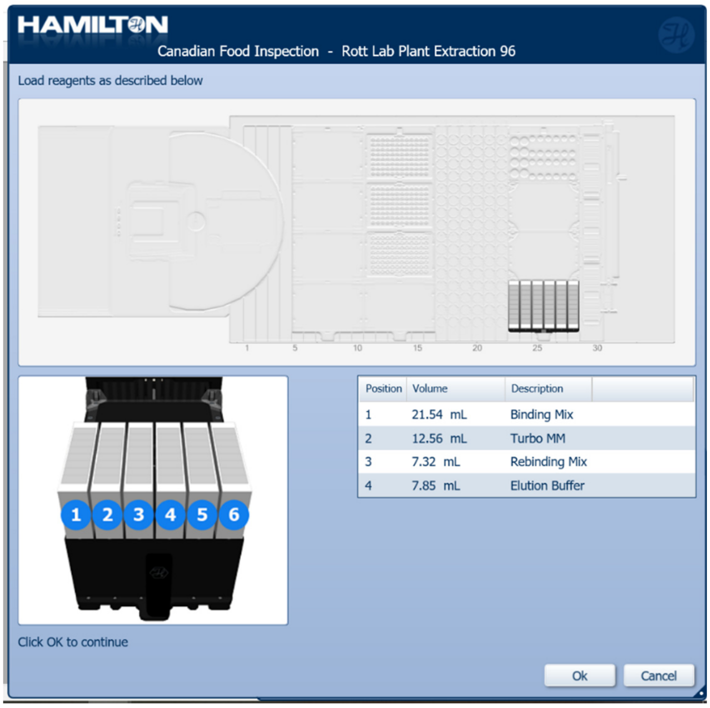

1.15 Load plates. 6 plates at positions 1-6. A single plate with tip comb at position 9.

*The position 9 plate only serves to hold a clean tip comb and can be re-used in subsequent extractions.

The plate at position 11 is the Sample plate.
*For automated sample transfer this plate will be empty, for manual sample transfer, wells will contain 600 µL cleared plant lysate. **Press OK**.

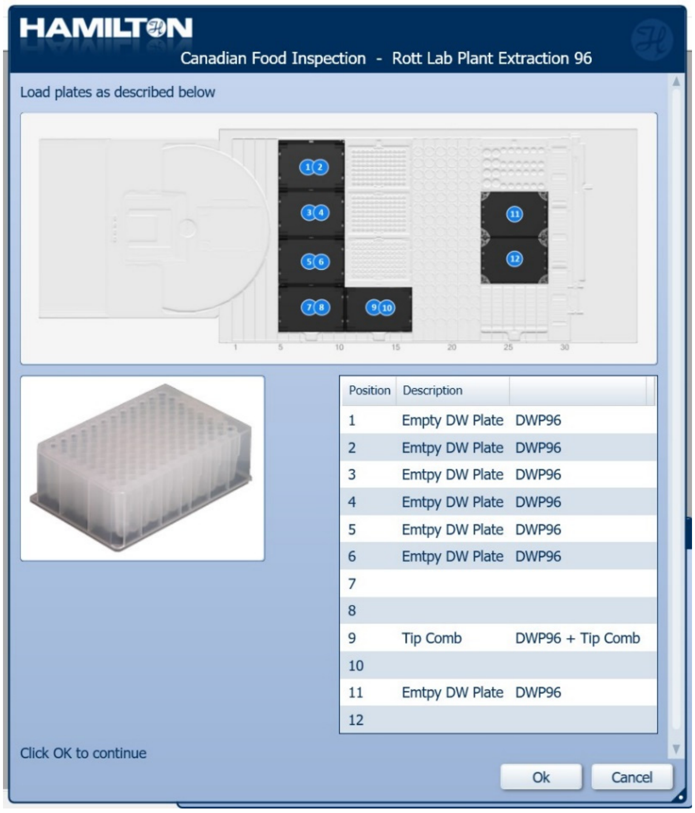

1.16 Load 1000 µL filter tips ≥ ‘Required number of tips’. A total of 3 racks (288 tips) can be loaded. Press OK.

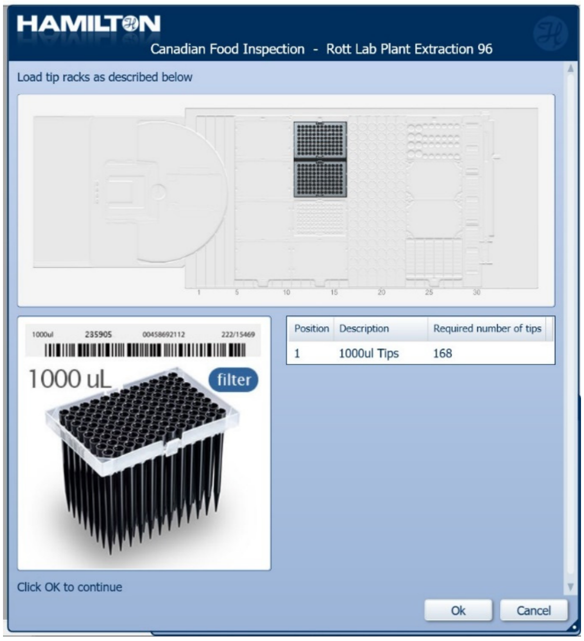

1.17 Load uncapped 5 mL tubes with recently centrifuged or previously transferred cleared plant lysate (if using the 50 mL tube method samples will have already been transferred to the sample plate in step 8.14), to the leftmost sample carrier as indicated. Sample 1 will represent the top position of the leftmost sample carrier. Subsequent samples will descend vertically and sample 16 will represent the bottom of that carrier. The top of the adjacent carrier will be sample 17 and so on. Press OK.

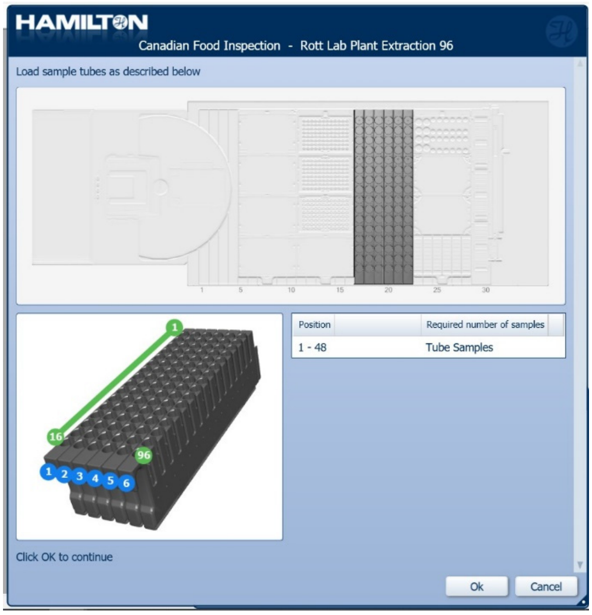

1.18 From the Tip Count window, indicate the tips available. Press OK. For runs where a full 96 samples with automated sample transfer will take place, the maximum tip number will be exceeded. To add more tips mid-method see the document.

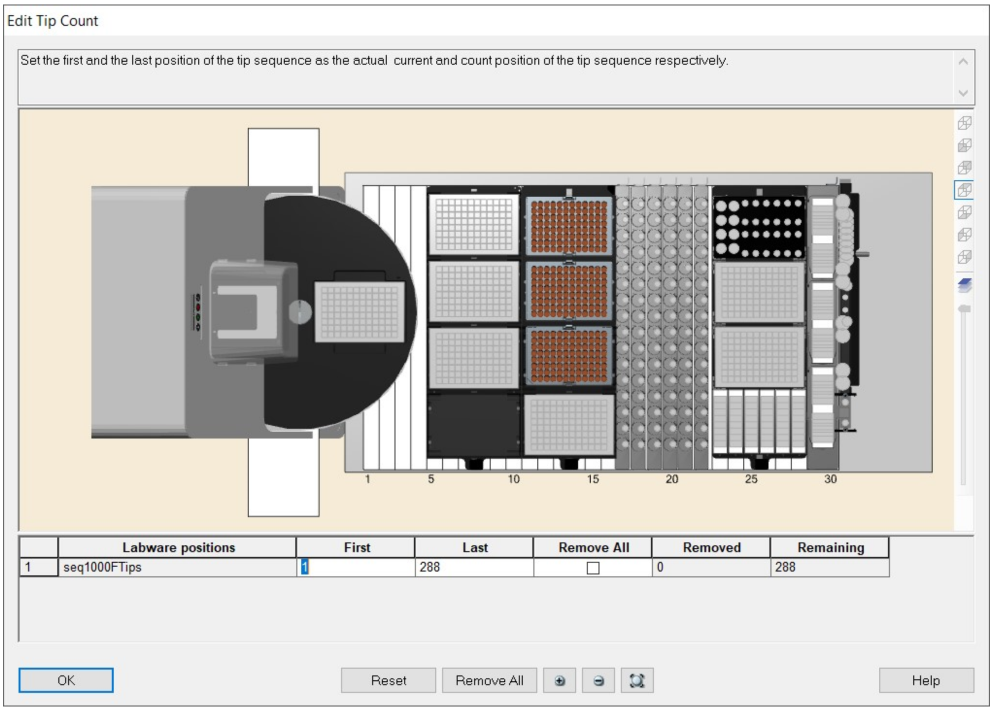

Type into the table or click, drag, and release in the diagram to select the tip wells to edit. A red tip well tells the machine that a tip is present, while a grey tip well tells it that there is no tip. Selecting a tip well will change it to the other state.

1.19 The final window before initialization and start of the automated method asks to ensure that the 96 Head is loaded on the Presto. Close the front cover of the Nimbus Presto. Press OK.

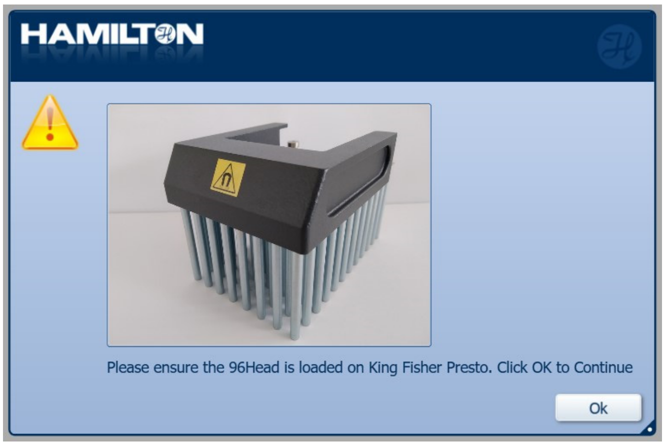

1.20 AUTOMATED STEPS OVERVIEW

Nimbus transfer of tip comb to Presto.

Sample plate preparation - Transfer of sample from 5 mL tubes to Sample plate. Addition of Binding Mix. Nimbus transport to Presto. Sample binding for ~12 mins, then resin collection.

Pre-wash 1 - Dispense wash. Transport. Resin washing for ~3mins, collection and drying for 1 min.

Nuclease Digestion - Dispense Turbo Master Mix. Transport. Sample nuclease treatment for 30mins at 37ºC. Addition of Rebinding Mix. Sample binding for ~12 mins and resin collection.

Post-wash 1, 2, 3 - Dispense wash. Transport. Resin washing for ~3mins each followed by collection. Resin drying for 2mins after final wash.

Elution - Dispense Elution Buffer. Transport. 5 min Elution of purified dsRNA.

*The duration of the automated portion of the method will depend on the number of samples run. A 48 sample extraction has a run time of ~2 hr.

1.21 At completion of the method, the Elution plate containing purified dsRNA will be placed at plate position 11 (Initial start position of the Sample plate). Press OK at the process complete window to close the program and unlock the front cover.

1.22 Remove KF Deep Well Elution plate.

Transfer 15 µL aliquots of each sample to a PCR Frame Plate for use in library preparation on the Hamilton Microlab STAR.

Seal the PCR FramePlate with Microseal B and place at –20 ºC.

Seal the KF Deep Well plate containing the remaining eluate with Microseal F and place at –80 ºC.

1.23 If performing the PvEV-1 assay. Transfer 3 µL aliquots to PCR strip tubes with dome caps.

Text S2. SAMPLE PLATE LAYOUT

Sample Carrier Position #	Sample Name	Sample Plate Well	Sample Carrier Position #	Sample Name	Sample Plate Well	Sample Carrier Position #	Sample Name	Sample Plate Well
1		A01	17		A03	33		A05
2		B01	18		B03	34		B05
3		C01	19		C03	35		C05
4		D01	20		D03	36		D05
5		E01	21		E03	37		E05
6		F01	22		F03	38		F05
7		G01	23		G03	39		G05
8		H01	24		H03	40		H05
9		A02	25		A04	41		A06
10		B02	26		B04	42		B06

11		C02
12		D02
13		E02
14		F02
15		G02
16		H02

27		C04
28		D04
29		E04
30		F04
31		G04
32		H04

43		C06
44		D06
45		E06
46		F06
47		G06
48		H06

Sample Carrier Position #	Sample Name	Sample Plate Well
49		A07
50		B07
51		C07
52		D07
53		E07
54		F07
55		G07
56		H07
57		A08
58		B08
59		C08
60		D08
61		E08
62		F08
63		G08
64		H08

Sample Carrier Position #	Sample Name	Sample Plate Well
65		A09
66		B09
67		C09
68		D09
69		E09
70		F09
71		G09
72		H09
73		A10
74		B10
75		C10
76		D10
77		E10
78		F10
79		G10
80		H10

Sample Carrier Position #	Sample Name	Sample Plate Well
81		A11
82		B11
83		C11
84		D11
85		E11
86		F11
87		G11
88		H11
89		A12
90		B12
91		C12
92		D12
93		E12
94		F12
95		G12
96		H12

Text S3. NIMBUS PRESTO PROTCOL

The Nimbus Presto integrates two components, the Nimbus which dispenses reagent solutions and transports plates, and the Presto which processes the samples as outlined in the BindIt file, NimbusPresto_RottLab96PlantExtraction_v1.bdz.

Nimbus transfer of tip comb to Presto.

Sample plate preparation - Transfer of 600 µL cleared sample lysate from 5 mL tubes to Sample plate. Addition of 370 µL Binding Mix. Nimbus transport to Presto.

Binding 5mins Fast mix speed then 1min pause, Loop count 2.

Binding Collect with collect count =3, collect time =1 sec.

Pre-Wash - Dispense 700 µL STE-18 wash. Nimbus transport to Presto.

Pre-Wash with Release beads, 1min Fast mix speed then 2 min pause.

Pre-Wash Collect with collect count =3, collect time =1 sec.

Dry 1min.

Nuclease Digestion - Dispense 200 µL Turbo Master Mix. Nimbus transport to Presto.

Nuclease with Release beads, 30 min medium mix speed at Block temperature 37°C.

Addition of Rebinding Mix – Nimbus transport from Presto to dispensing platform. Addition of 100 µL Rebinding Mix. Nimbus transport to Presto.

Rebinding 5 min Fast mix speed then 1 min pause, Loop count 2.

Rebinding Collect with collect count =3, collect time =1 sec.

Post-Wash 1 - Dispense 700 µL STE-18 wash. Nimbus transport to Presto.

Post-Wash 1 with Release beads, 1 min Fast mix speed then 2 min pause.

Post-Wash 1 Collect with collect count =3, collect time =1 sec.

Post-Wash 2 - Dispense 700 µL ST-80 wash. Nimbus transport to Presto.

Post-Wash 2 with Release beads 15 sec Bottom mix, 1 min Fast mix speed, 2 min pause.

Post-Wash 2 Collect with collect count =3, collect time =1s.

Post-Wash 3 - Dispense 700 µL ST-80 wash. Nimbus transport to Presto.

Post-Wash 3 with Release beads 15 sec Bottom mix, 1 min Fast mix speed, 2 min pause.

Post-Wash 3 Collect with collect count =3, collect time =1 sec.

Dry 2 mins.

Elution – Dispense 110 µL Elution Buffer. Nimbus transport to Presto.

Elution with Release beads, 5 min Fast mix speed.

Elution Collect with collect count =5, collect time =30 sec.

Text S4. PREPARATION OF REAGENTS

<u>1M TRIS Solution, pH 8.0 (500 mL)</u>

- Add 60.55 g Tris in 300 mL Type 1 H_2O
- Stir vigorously
- Add concentrated HCl dropwise to bring pH to 8.0 ± 0.05
- Wait until solution reaches to room temperature, then measure the pH and adjust if needed
- Bring volume up to 500 mL with Type 1 H_2O
- Autoclave
- Store at room temperature

<u>1M TRIS Solution, pH 8.3 (500 mL)</u>

- Add 60.55 g Tris in 300 mL Type 1 H_2O
- Stir vigorously
- Add concentrated HCl dropwise to bring pH to 8.3 ± 0.05
- Wait until solution reaches to room temperature, then measure the pH and adjust if needed
- Bring volume up to 500 mL with Type 1 H_2O
- Autoclave
- Store at room temperature

<u>0.5 M EDTA Solution, pH 8.0 (200 mL)</u>

- Add 29.22 g of EDTA to approximately 120 mL of Type 1 H_2O.
- Stir vigorously.
- Adjust the final pH to 8.0 ± 0.05 using NaOH.
- Bring the final volume up to 200 mL with Type 1 H_2O.
- Transfer to a reagent bottle and autoclave.
- Store at room temperature indefinitely.

<u>10X STE Buffer (500 mL)</u>

Fill a beaker with approximately 300 mL Type 1 H_2O and add the following:

- 50 mL 1M Tris, pH 8.0
- 29.22 g NaCl
- 10 mL 0.5M EDTA, pH 8.0 solution
- Bring volume up to 500 mL with Type 1 H_2O
- Autoclave
- Store at room temperature

<u>10X ST Buffer (500 mL)</u>

Fill a beaker with approximately 300 mL Type 1 H_2O and add the following:

- 50 mL 1M Tris, pH 8.0
- 29.22 g NaCl
- Bring volume up to 500 mL with Type 1 H_2O
- Autoclave
- Store at room temperature

<u>Hybrid Extraction Buffer A (500 mL)</u>

Fill a beaker with approximately 300 mL of Type 1 H_2O and add:

- 100 mL 1M Tris, pH 8.3
- 10 g PVP 40 000 **(dust mask)**
- 5 g PEG 8000 **(dust musk)**
- 9.58 g NaCl
- 0.1 g sodium sulfite
- 10 mL 0.5 M EDTA solution
- 0.5 mL Triton
- 2.5 mL Tween 20
- Stir using a large magnetic stir bar
- Allow to dissolve
- Bring volume up to 500 mL with Type 1 H_2O
- Store at 4°C for up to 3 months

<u>STE-18 (1X STE Buffer + 18% ethanol) (500 mL)</u>

- Add 50 mL 10X STE buffer to 90 mL 100% ethanol
- Bring up to 500 mL with Type 1 H_2O
- Store at room temperature up to 3 months

<u>ST-80 (1X ST Buffer + 80% ethanol) (500 mL)</u>

- Add 50 mL 10X ST buffer to 400 mL 100% ethanol
- Bring up to 500 mL with Type 1 H_2O
- Store at room temperature up to 3 months

<u>10mM Tris pH 8.0 (200 mL)</u>

- Add 2 mL 1M Tris pH 8.0 solution to 198 mL Type 1 H_2O
- Autoclave
- Store at room temperature
