# Supplementary material for: A Validation Study of a High-Throughput Sequencing Workflow for the Detection of Grapevine Viruses and Viroids
Source: Pathogens. 2026 Sep 21;15(9):998. doi: 10.3390/pathogens15090998 (PMC13610249; doi:10.3390/pathogens15090998)
Supplement: Supplementary file 1 [file pathogens-15-00998-s001.zip › Supplemental Document S3. HTS Library from dsRNA Method.pdf]

Supplemental Document S3: HTS Library from dsRNA Method

Note: Cross-contamination is most likely to occur during the first manual sample-handling steps

1.1 For each set of libraries, record the sample names and RAP indices to be used.

1.2 Preload 50µL, 300µL and 1000µL tips in tip carrier based on the number of sample libraries to be created, 24, 32 or 48. (Refer to **Text S6 – Table S1)**.

1.3 Remove the required library reagents from -20ºC and allow to thaw at room temperature. Store on ice until ready to use. Record lot numbers on the checklist.

1.4 Remove dsRNA extracts from the freezer and let thaw.

1.5 Transfer 15µL dsRNA to the appropriate wells of a new PCR FramePlate. Load samples starting with column 1 and continue with consecutive columns as needed. Seal plate with BioRad Microseal B until ready to load or store overnight at -20°C.

1.6 Prepare fresh, 125 mL 80% EtOH on each day.

1.7 Turn on the robotics with See **Text S5 – Figures S5 and S6** for switch locations.

1.8 Open TruSeq Stranded dsRNA program with Hamilton Run Method Manager. Select the appropriate Example Plate File according to number of samples and indices to be used. For routine runs, ensure the following settings: Fragmentation - 8, In-kit controls - No, Adapter - RAP, Pooling - No. Ensure controller boxes are on. Press OK.

Run Settings

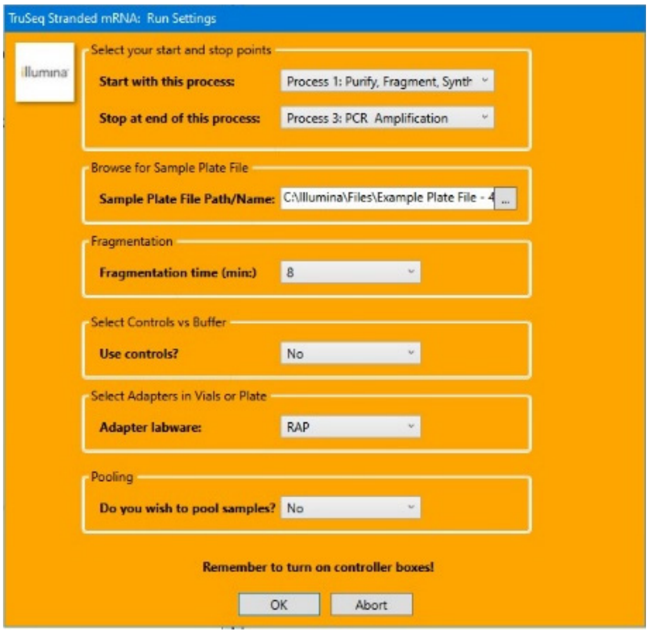

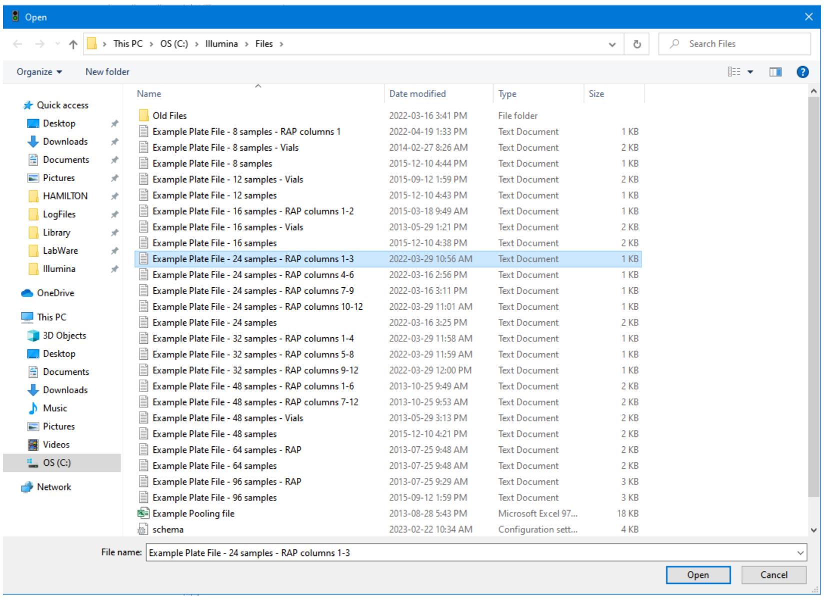

1.9 From the Required Resources window, load MIDI plates and HSPs (PCR FramePlates) as indicated to Stacker carrier. Load filter tips to tip carriers if not done previously. See **Text S5 – Figure S7** for deck layout.

1.10 Add required amount of FSA+ SSII (refer to **Text S6 – Table S1** for amounts depending on number of samples).

1.11 Vortex all reagents. Spin down. Load tubes onto the positions as indicated in the bottom box (labeled Vial Carrier – Track 33) of the "Reagent 32-vial carrier". Orient the reagent tubes to ensure barcodes are visible to the bar code scanner. (Refer to **Text S6 – Table S1** for amounts depending on number of samples).

Required Resources

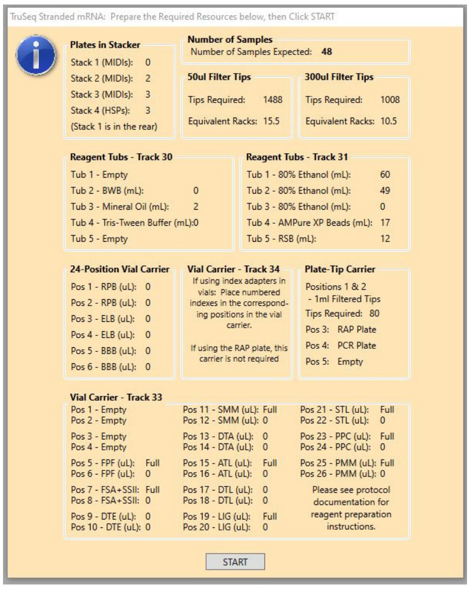

1.12 Transfer 3mL of mineral oil to reagent tub. Load 60mL reagent tub as indicated by Track 30.

1.13 Transfer indicated amounts of freshly prepared 80% ethanol, AMPure XP beads (or Illumina Purification Beads) and RSB into 60mL reagent tubs. (refer to **Text S6 – Table S1** for amounts depending on number of samples). Load reagent tubs as indicated by Track 31.

1.14 Instead of loading the RAP plate, load an empty PCR FramePlate to position 3 of the Plate-Tip carrier. The RAP plate will be added later to the method. If already thawed, the sealed RAP plate can be kept at 4°C until needed.
*Ensure an appropriate concentration of the RAP plate is utilized (refer to Text S6) ;
for dsRNA extracts using the Nimbus Presto method – 1/3 diluted RAP,

1.15 Next, load the sample plate containing 15µL dsRNA to position 4 of the Plate-Tip carrier.

1.16 Press START and record starting time or set a timer for 2.5 hours (24 samples) or 3.5 hours (48 samples) for the next manual handling step.

1.17 Indicate location of tips within tip racks on map. Press OK.

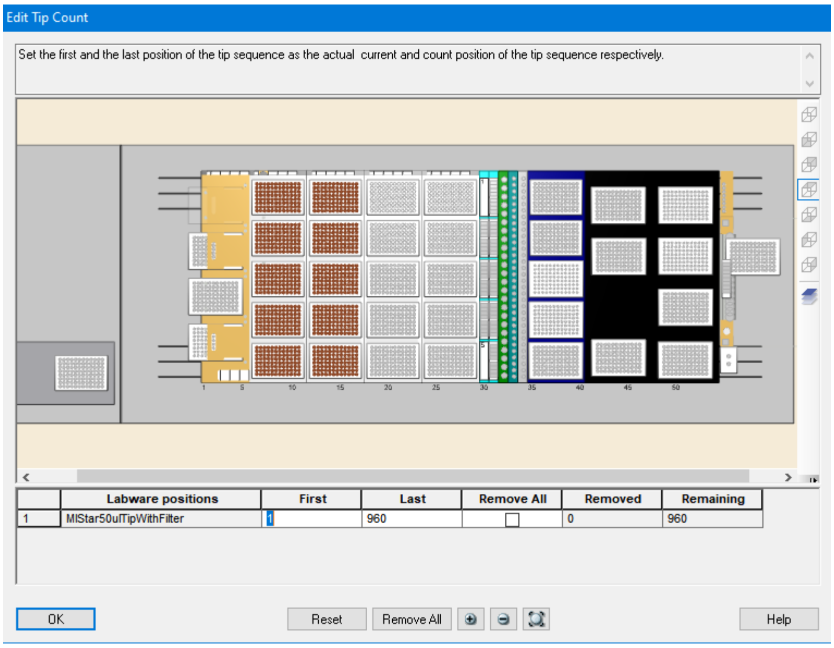

Click, drag, and release to select the tip wells to edit. A red tip well tells the machine that a tip is present, while a grey tip well tells it that there is no tip. Selecting a tip well will change it to the other state.

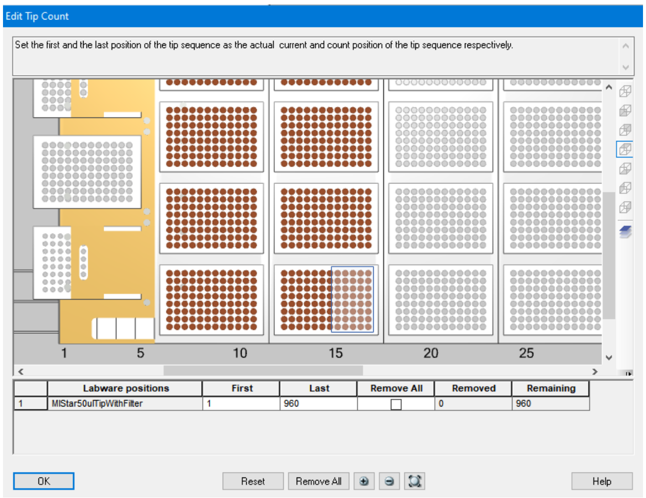

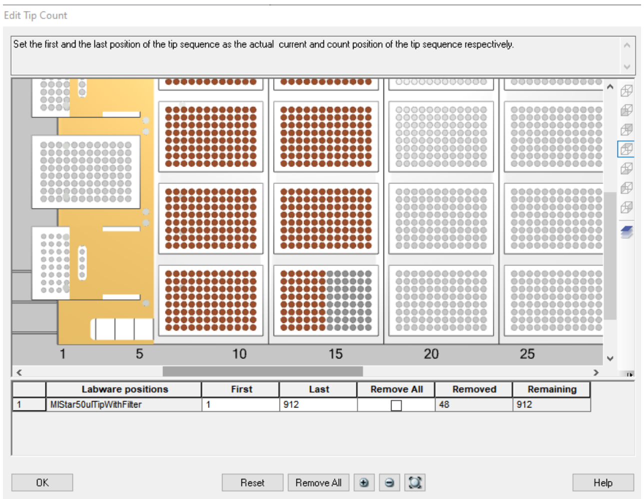

Each tip type/size is indicated on a separate dialogue. The tip being indicated is shown in the Labware Positions box (circled in green on the diagram). The tip dialogues occur in order of ascending volume (50µL, 300µL, 1000µL).

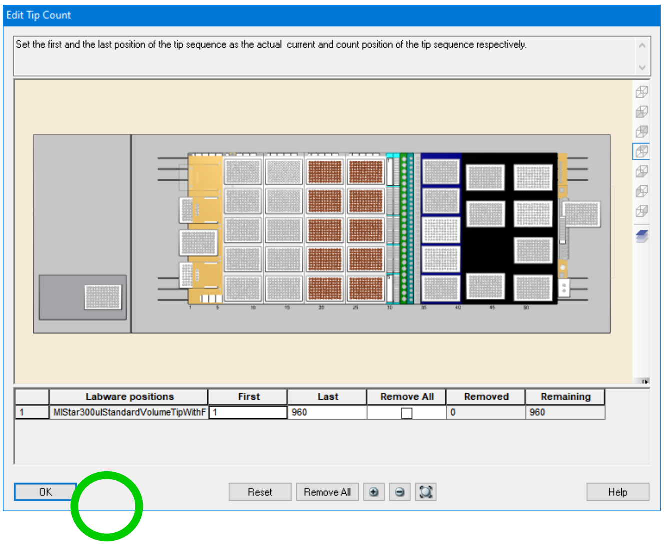

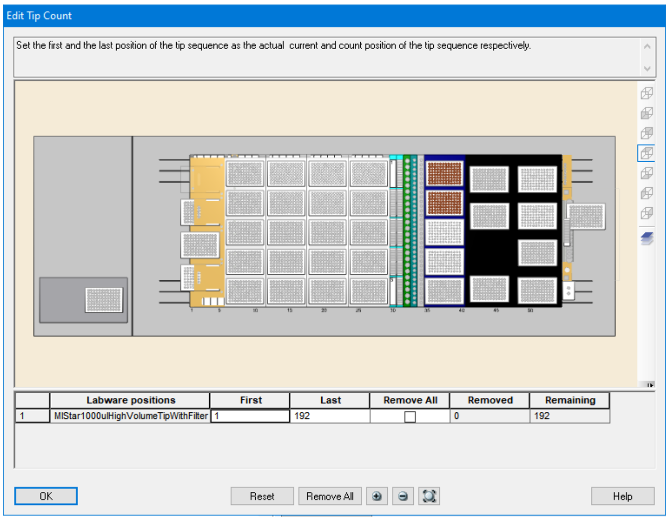

1.18 Press 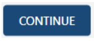 on ‘verify plate number in stack’ dialogue. A message may appear warning that not enough tips are loaded. Press OK. the user will be prompted to add more tips later (see step 8.26)

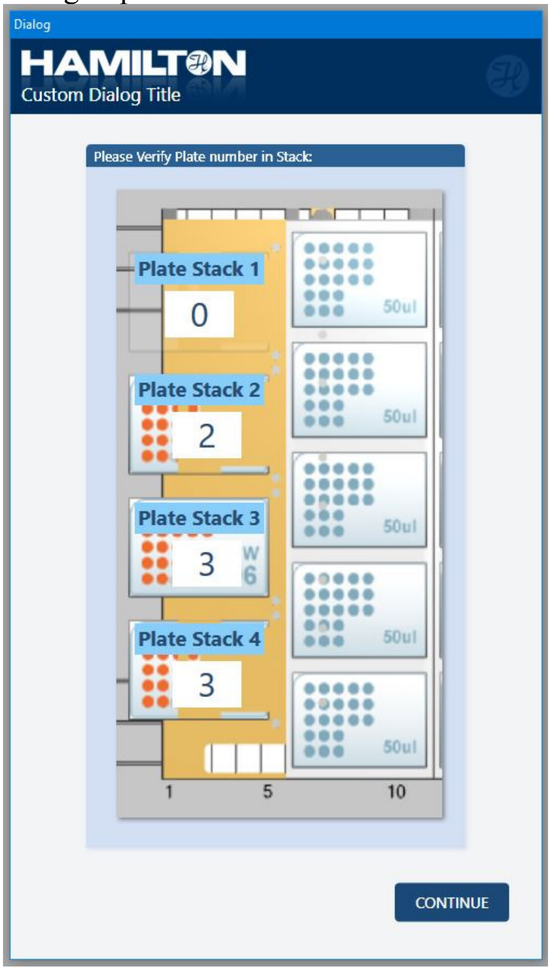

1.19 Leave carriers in the “out” position on the machine. Carriers will be autoloaded into the STAR and applicable barcodes scanned. If carriers are pushed in, the following dialogue will appear for every carrier pushed in. If this dialogue occurs, press Yes. The robotics will push each carrier out, then retract them to read the barcode.

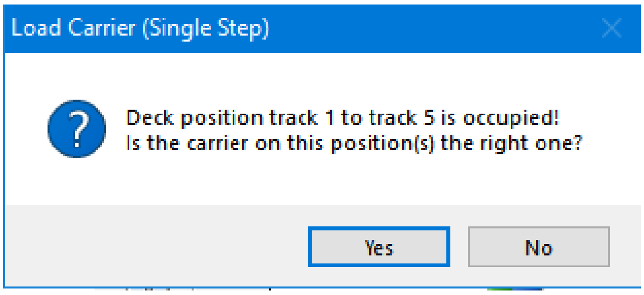

1.20 Verify the plates as indicated by the Plate-Tip carrier dialogue window. Press 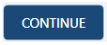. This will initiate the automated procedure, beginning with Process 1 (Purify, Fragment, Synthesize).

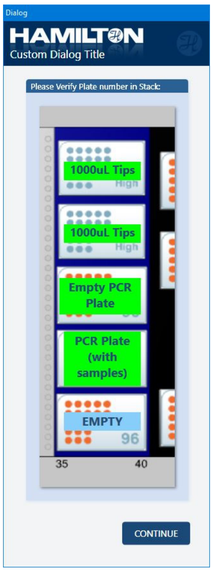

The higher-level steps of the procedure will be displayed in the “status window”.

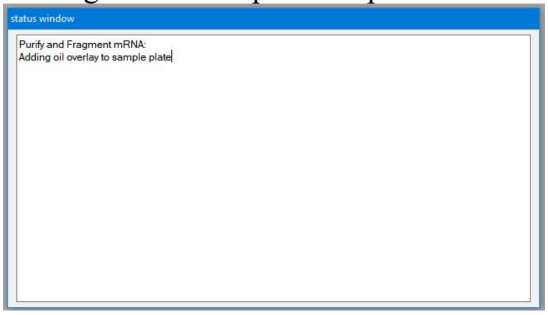

More detailed information will appear in the Trace View window.

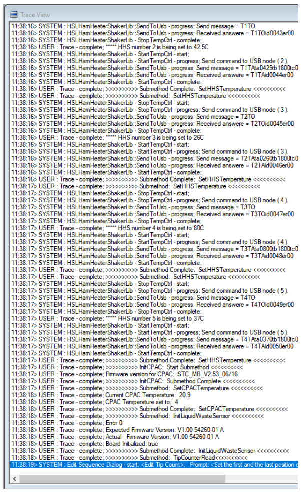

1.21 Prior to completion of **PROCESS 1**, the user will be required to spin down the sample MIDI plate. At this step the carrier will unload. Remove MIDI plate and seal plate with Microseal B. Centrifuge at 500 x g for 30 seconds.

1.22 Remove the seal and return MIDI to position 5 of the Plate-Tip carrier.

1.23 Add the appropriate RAP plate. 1/3 diluted RAP – for Nimbus Presto prepared dsRNA extracts, Neat concentration RAP – for manual cellulose prepared dsRNA extracts. Remove the seal or pierce the foil of the RAP plate wells to be used, with the bottom of fresh PCR strip tubes.

1.24 Load RAP plate to position 3 of the Plate-Tip carrier as shown below. ENSURE THAT RAP PLATE IS IN THE CORRECT ORIENTATION. (A1-back left). Press Continue.

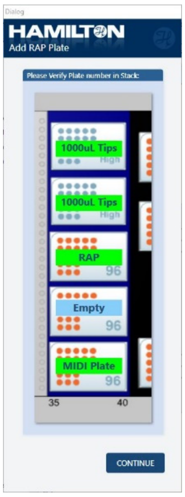

1.25 Turn on and preheat the thermocycler

1.26 Offload sample plate from carrier for PCR amplification. Seal plate with Microseal A. Centrifuge the plate at 500 x g for 30 seconds. Load plate into thermocycler and start program. 98ºC for 30 seconds + 15 cycles [98ºC for 10 seconds + 60ºC for 30 seconds + 72ºC for 30 seconds] + 72ºC for 5 minutes, then hold at 4ºC.

1.27 Remove mineral oil tub, RAP plate and reagents tubes from the carriers. Replace lids and return reagent tubes and sealed RAP plate to -20ºC if necessary.

1.28 Load additional 50µL tips as necessary. Adjust 50µL tip location map for added tips. Press OK.

1.29 Centrifuge amplified PCR plate at 500 x g for 30 seconds, then remove seal. Return plate to position 4 of the Plate-Tip carrier and press “OK” in the dialogue to continue with the post-PCR automated clean-up of **PROCESS 3**.

1.30 END OF PROCESS 3. Program will finish and the plate containing the sample libraries (TSP1 plate) will be unloaded. Continue with library quantification or seal plate with Microseal B and place at -20ºC. Label plate and seal as 'undiluted libraries' with Library date/ID. Sequencing of libraries within 7 day is preferred, however libraries stored for longer periods can be successfully sequenced.

1.31 Remove reagents tubes Remove and save unused tips.

2.0 Library Quantification with Qubit 3, 4 or Flex Fluorometer and Normalization

2.1 Prepare Qubit working buffer by adding Qubit dsDNA HS buffer into a 15 mL tube and Qubit dsDNA HS reagent. (refer to **Text S6 – Table S1** for amounts depending on number of samples). Vortex well.

2.2 Prepare a Qubit assay tube (Qubit 3 or 4) or a Qubit strip tube (Qubit Flex) each for standard 1 and standard 2. Add 190 µL of Qubit working buffer and 10 µL of the appropriate standard. Vortex well or shake at 1800rpm for 1 minute. Incubate 2 minutes.

2.3 Prepare Qubit assay tubes or Qubit strip tubes for sample libraries. Add 197 µL of Qubit working buffer and 3 µL of the appropriate library to each tube. Vortex well or shake at 1800rpm for 1 minute. Incubate 2 minutes.

2.4 Spin all samples briefly to remove air bubbles.

2.5 Touch the screen to power on the Qubit fluorometer. From the home screen select "dsDNA High Sensitivity".

2.6 Select "Read Standards". Insert standard 1 and press "Run Standard". Wait for the Qubit to display and/or approve the concentration. Repeat for standard 2.

2.7 Select "Run Samples". Adjust volume to 3 µL and Output Sample Units to "ng/mL".

2.8 Read samples and record values in the sample library table of form CPH136.

2.9 Open HTS Library Log.xlsx and enter Qubit readings. The formula embedded will automatically calculate the sample molarity.

2.10 Determine an appropriate concentration for sample normalization, usually 4nM or 2nM.

2.11 Copy sample names and molarity values.

2.12 Normalize individual sample libraries by diluting with RSB. Prepare in a labelled PCR plate (max 50 uL) or in 0.5 mL microcentrifuge tubes (max 500 uL). Ensure a minimum sample volume of 15 uL. Store libraries at -20ºC.

*If 1nM normalization is selected, dilute libraries directly with RSB with Tween 20.

Text S5: Robotics diagrams

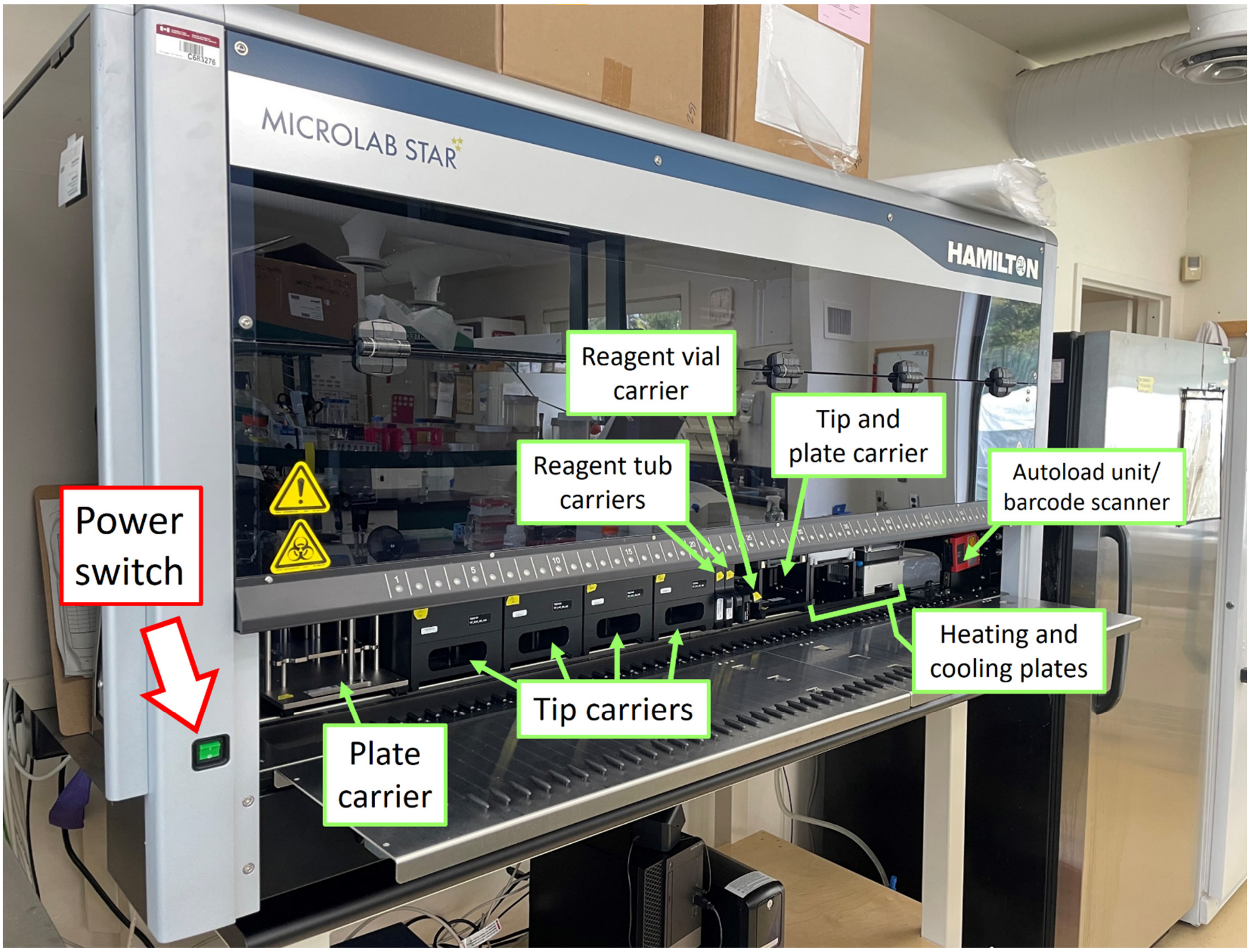

Figure S5. Microlab STAR robotics front view.

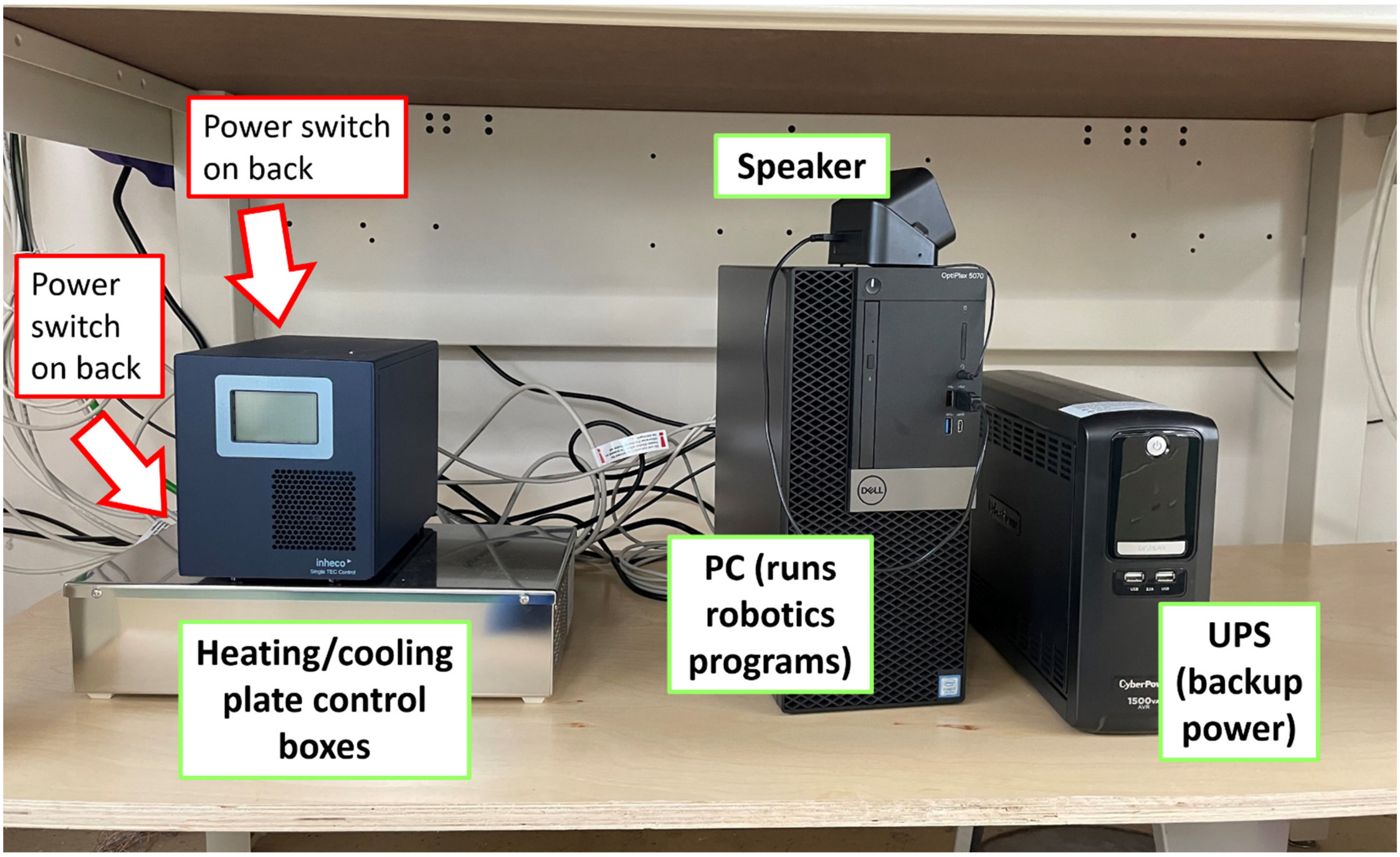

Figure S6. Robotics controller boxes and computer setup below MICROLAB STAR unit.

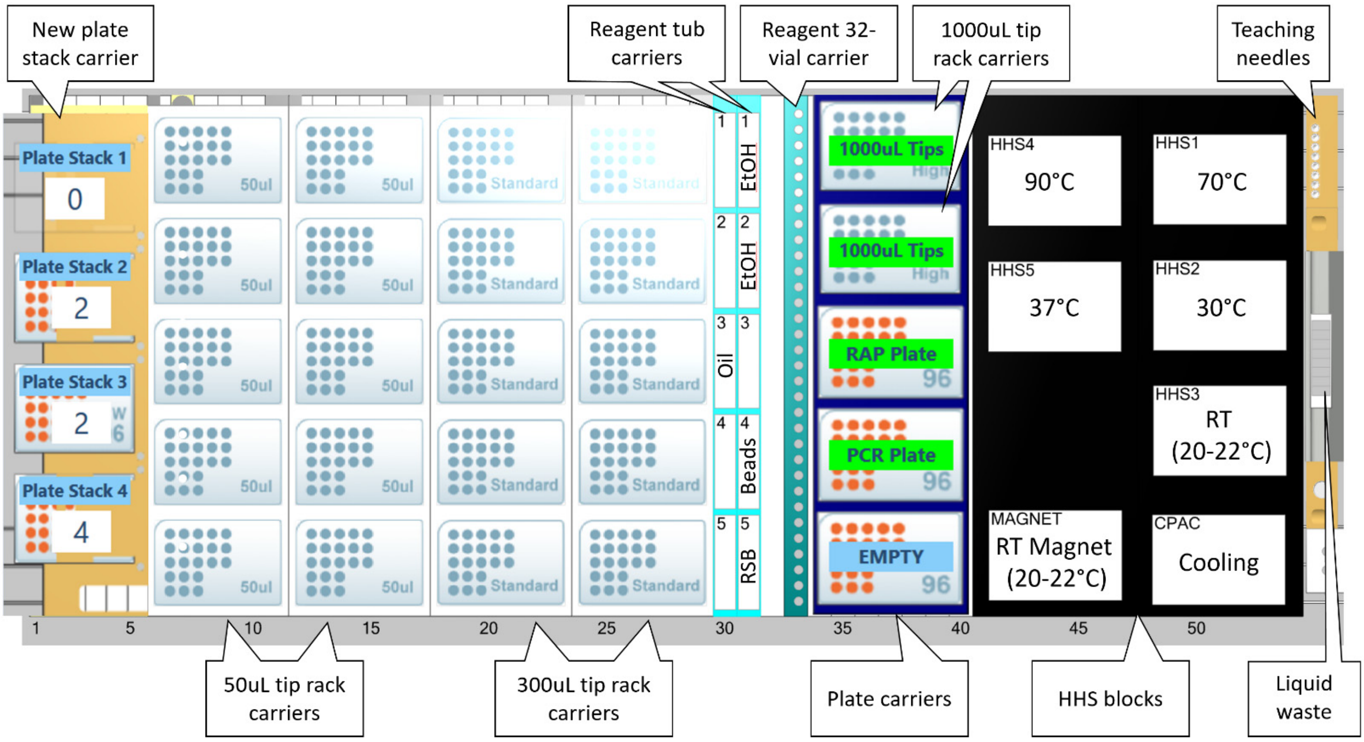

Figure S7. Deck diagram of MICROLAB STAR robotics.

Text S6: PREPARATION OF REAGENTS AND CONSUMABLES

80% Ethanol (125mL)

- Add 100 mL 100% ethanol
- Bring up to 125 mL with sUP H_2O

Prepare fresh each day

70% Ethanol (950mL) – for cleaning

- Add 700 mL 95% ethanol
- Bring up to 950 mL with sUP H_2O

sUP H_2O (1L)

- Autoclave 1L of ultra-purified water

FSA+SSII

- Add 50 µL SuperScript II RT into a full FSA (First Strand Synthesis Act D Mix) tube for a 48 sample run. If you are not using the entire contents, the remainder can be stored at -20ºC or the preferred method is to only make up the volume of FSA+SSII required, by adding SuperScript II at a ratio of 1µL SuperScript II for each 9µl First Strand Synthesis Act D Mix. Label the First Strand Synthesis Act D Mix tube that the SuperScript II has been added. Mix, and centrifuge briefly. Ensure the FSA +SSII mix is in the native (or equivalent) tube for loading on to the vial carrier.

RNA Adapter Plate (RAP) – 1/3 Diluted

- Remove the RAP plate from the -20ºC freezer and allow to thaw at room temperature. Centrifuge at 500 x g for 30sec. Pierce the foil seal of the RAP plate wells with fresh PCR strip tubes or a suitable PCR plate. To each 10µL UDI adapter add 20µL RSB. Mix well by pipetting. From each 30µL total volume, aliquot 14µL to a new PCR FramePlate in the **SAME CONFIGURATION** as the initial RAP plate. From one original RAP there is enough volume to prepare two – 1/3 diluted RAP. Seal and label newly created RAP plates with the date aliquoted, 1/3 dilution factor, UDI range and original lot #.

Table S1. Required Resources for complete TruSeq Stranded dsRNA Library Preparation

Required tips & reagents	Lot #	Steps	Number of Samples: 24	Number of Sample: 32	Number of Samples: 48
Tips					
50µL filter tips		8.2	7.8 racks	10.3 racks	15.5 racks
300µL filter tips		8.2	5.3 racks	7 racks	10.5 racks
1000µL filter tips		8.2	80 tips	80	80 tips
Library Reagents					
80% Ethanol (mL)		8.14	54	77 (60 +17)*	109 (60 + 49)*
AMPure XP / IPB (mL)		8.14	11	13	17
RSB (mL)		8.14	8	9	12
FPF (µL)		8.12	505	665	Full (1010)
FSA(µL)		8.12	225	297	Full (460)
SSII (µL)		8.12	25	33	50
SMM (µL)		8.12	559.2	725.6	Full (1040)
ATL (µL)		8.12	362.2	464.6	Full (680)
LIG (µL)		8.12	119.4	144.2	Full (270)
STL (µL)		8.12	144.8	186.4	Full (480)
PPC (µL)		8.12	174.8	216.4	Full (320)
PMM (µL)		8.12	671.4	880.2	Full (1280)
Quantification					
Qubit dsDNA HS buffer (µL)		9.1	8557	10149	13532
Qubit dsDNA HS Reagent (µL)		9.1	43	51	68

***Note: the tubs hold a maximum of 60 ml. Divide the ethanol in 2 tubs with 60 mL in the first and the remaining volume in the other.**
