# Supplementary material for: A Validation Study of a High-Throughput Sequencing Workflow for the Detection of Grapevine Viruses and Viroids
Source: Pathogens. 2026 Sep 21;15(9):998. doi: 10.3390/pathogens15090998 (PMC13610249; doi:10.3390/pathogens15090998)
Supplement: Supplementary file 1 [file pathogens-15-00998-s001.zip › Supplemental Figure S1.pdf]

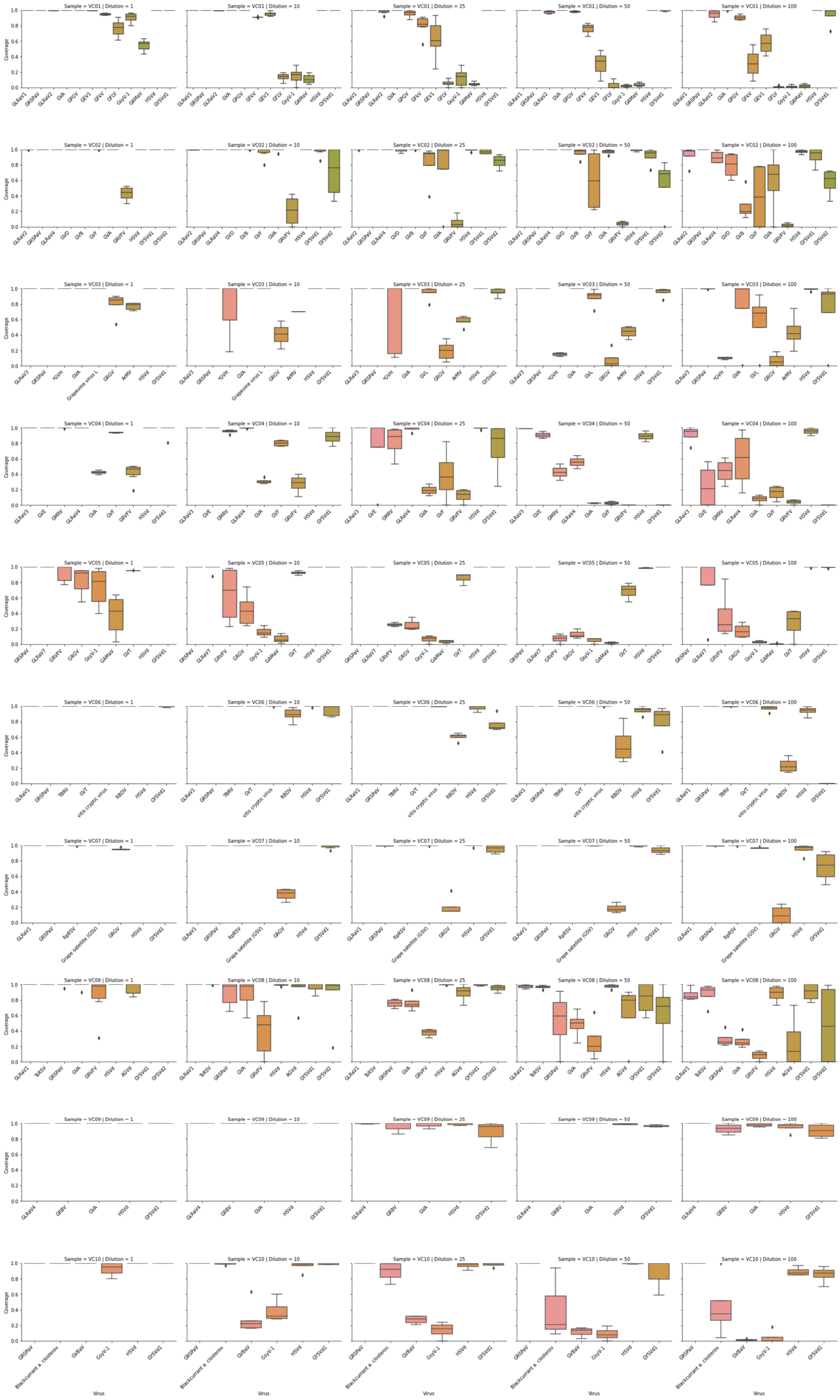

Sample = VC01 | Dilution = 1
Sample = VC01 | Dilution = 10
Sample = VC01 | Dilution = 25
Sample = VC01 | Dilution = 50
Sample = VC01 | Dilution = 100
Sample = VC02 | Dilution = 1
Sample = VC02 | Dilution = 10
Sample = VC02 | Dilution = 25
Sample = VC02 | Dilution = 50
Sample = VC02 | Dilution = 100
Sample = VC03 | Dilution = 1
Sample = VC03 | Dilution = 10
Sample = VC03 | Dilution = 25
Sample = VC03 | Dilution = 50
Sample = VC03 | Dilution = 100
Sample = VC04 | Dilution = 1
Sample = VC04 | Dilution = 10
Sample = VC04 | Dilution = 25
Sample = VC04 | Dilution = 50
Sample = VC04 | Dilution = 100
Sample = VC05 | Dilution = 1
Sample = VC05 | Dilution = 10
Sample = VC05 | Dilution = 25
Sample = VC05 | Dilution = 50
Sample = VC05 | Dilution = 100
Sample = VC06 | Dilution = 1
Sample = VC06 | Dilution = 10
Sample = VC06 | Dilution = 25
Sample = VC06 | Dilution = 50
Sample = VC06 | Dilution = 100
Sample = VC07 | Dilution = 1
Sample = VC07 | Dilution = 10
Sample = VC07 | Dilution = 25
Sample = VC07 | Dilution = 50
Sample = VC07 | Dilution = 100
Sample = VC08 | Dilution = 1
Sample = VC08 | Dilution = 10
Sample = VC08 | Dilution = 25
Sample = VC08 | Dilution = 50
Sample = VC08 | Dilution = 100
Sample = VC09 | Dilution = 1
Sample = VC09 | Dilution = 10
Sample = VC09 | Dilution = 25
Sample = VC09 | Dilution = 50
Sample = VC09 | Dilution = 100
Sample = VC10 | Dilution = 1
Sample = VC10 | Dilution = 10
Sample = VC10 | Dilution = 25
Sample = VC10 | Dilution = 50
Sample = VC10 | Dilution = 100
Coverage
Virus

Supplementary Figure S1. Distribution of viral genome coverage values in all dilutions for virus composite (VC) samples. Box plots for undiluted samples, the minimum replicate n=4 (VC10); for 10-fold-diluted samples, the minimum n=3 (VC03); for 25-fold-diluted samples, the minimum n=3 (VC05 & VC09); for 50-fold-diluted samples, the minimum n=2 (VC04 & VC09); for 100-fold-diluted samples, the minimum n=4 (all samples) for the HTS datasets. Panels correspond to VC01-VC10 arranged in five rows and four columns, with the viruses and viroids present in each VC shown on the x-axis. The y-axis shows genome coverage (0.0-1.0). *GVH = (GVH/GVM specific weight).
